# Cohesin mutations alter DNA damage repair and chromatin structure and create therapeutic vulnerabilities in MDS/AML

**DOI:** 10.1172/jci.insight.142149

**Published:** 2021-02-08

**Authors:** Zuzana Tothova, Anne-Laure Valton, Rebecca A. Gorelov, Mounica Vallurupalli, John M. Krill-Burger, Amie Holmes, Catherine C. Landers, J. Erika Haydu, Edyta Malolepsza, Christina Hartigan, Melanie Donahue, Katerina D. Popova, Sebastian Koochaki, Sergey V. Venev, Jeanne Rivera, Edwin Chen, Kasper Lage, Monica Schenone, Alan D. D’Andrea, Steven A. Carr, Elizabeth A. Morgan, Job Dekker, Benjamin L. Ebert

**Affiliations:** 1 Department of Medical Oncology, Dana-Farber Cancer Institute, Boston, Massachusetts, USA.; 2 Cancer Program, Broad Institute, Cambridge, Massachusetts, USA.; 3 Program in Systems Biology, Department of Biochemistry and Molecular Pharmacology, University of Massachusetts Medical School, Worcester, Massachusetts, USA.; 4 Department of Radiation Oncology, Dana-Farber Cancer Institute, Boston, Massachusetts, USA.; 5 Faculty of Biological Sciences, University of Leeds, Leeds, United Kingdom.; 6 Department of Pathology, Brigham and Women’s Hospital, Boston, Massachusetts, USA.; 7 Howard Hughes Medical Institute, Chevy Chase, Maryland, USA.

**Keywords:** Hematology, Oncology, Epigenetics, Leukemias, Mouse models

## Abstract

The cohesin complex plays an essential role in chromosome maintenance and transcriptional regulation. Recurrent somatic mutations in the cohesin complex are frequent genetic drivers in cancer, including myelodysplastic syndromes (MDS) and acute myeloid leukemia (AML). Here, using genetic dependency screens of stromal antigen 2–mutant (*STAG2*-mutant) AML, we identified DNA damage repair and replication as genetic dependencies in cohesin-mutant cells. We demonstrated increased levels of DNA damage and sensitivity of cohesin-mutant cells to poly(ADP-ribose) polymerase (PARP) inhibition. We developed a mouse model of MDS in which *Stag2* mutations arose as clonal secondary lesions in the background of clonal hematopoiesis driven by tet methylcytosine dioxygenase 2 (*Tet2*) mutations and demonstrated selective depletion of cohesin-mutant cells with PARP inhibition in vivo. Finally, we demonstrated a shift from STAG2- to STAG1-containing cohesin complexes in cohesin-mutant cells, which was associated with longer DNA loop extrusion, more intermixing of chromatin compartments, and increased interaction with PARP and replication protein A complex. Our findings inform the biology and therapeutic opportunities for cohesin-mutant malignancies.

## Abstract 2

We developed models of cohesin-mutant myelodysplastic syndromes and acute myeloid leukemia and demonstrated a shift from STAG2- to STAG1-cohesin complexes, increased DNA damage, and sensitivity to PARP inhibition.

## Graphical abstract

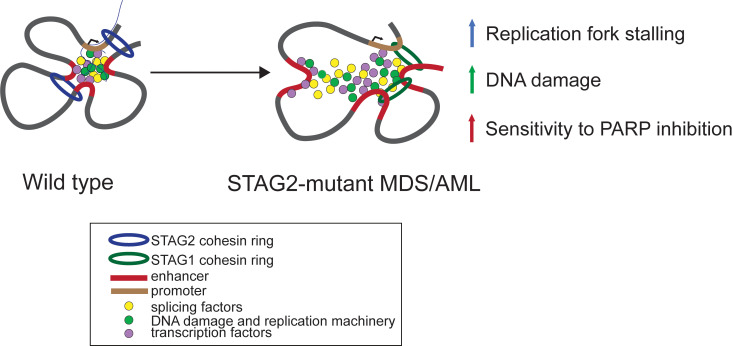

## Introduction

The cohesin complex is a multimeric protein complex that forms a ring structure around DNA molecules and plays multiple key roles in spatial organization of eukaryotic genomes. Cohesin proteins are involved in several essential cellular functions, including sister chromatid cohesion, chromatin loop organization, transcriptional activation, and DNA replication and damage repair, among others (reviewed in ref. 1). More recently, the cohesin complex was identified as one of the most frequently mutated protein complexes in cancer, including myeloid malignancies, glioblastoma, breast cancer, bladder cancer, and Ewing sarcoma (2, 3). The mechanisms by which cohesin mutations cause cellular transformation are unknown, and currently no therapies are known to exhibit selective efficacy in cohesin-mutant cancers.

Myelodysplastic syndromes (MDS) and acute myeloid leukemia (AML) are clonal diseases of mutated hematopoietic stem and progenitor cells (HSPCs) characterized by abnormal differentiation and proliferation caused by somatic mutations in genes encoding transcription factors, epigenetic regulators, chromatin modifiers, and splicing factors (4, 5). The core components of the cohesin complex, stromal antigen 2 (*STAG2*), structural maintenance of chromosomes 1 (*SMC1*), *SMC3*, and *RAD21*, as well as its modulators, *PDS5* and NIPBL cohesin loading factor (*NIPBL*), are collectively mutated in 13% of patients with de novo AML, 21% of patients with secondary AML, and 11% of patients with MDS, where they are associated with poor overall survival (5–10). Mutations in the cohesin genes are nearly always mutually exclusive, heterozygous, predicted loss-of-function (LOF) lesions, which are thought to be acquired early during the progression from clonal hematopoiesis of indeterminate prognosis to MDS (4, 11). Targeted inactivation of *Smc3* or *Stag2*, and overexpression of mutant cohesin genes in WT mouse and human HSPCs, have been previously studied (12–15), but no cohesin-mutant models currently exist that recapitulate the natural evolution of cohesin-mutant myeloid disease in the context of clonal hematopoiesis.

Two cohesin complexes are known to co-occur in somatic vertebrate cells, each containing the core components SMC1A, SMC3, and RAD21, and alternatively including STAG2 or the less abundant STAG1 (16). STAG2- and STAG1-containing complexes associate with centromeres and telomeres, respectively, but their sister chromatid cohesion-independent functional differences are not fully understood (1). *STAG2* mutations account for over 85% of cohesin mutations in MDS, whereas *STAG1* is rarely mutated in MDS or AML (6, 7, 10, 17). The mechanism underlying clonal expansion of these driver mutations is unlikely related to defects in sister chromatid cohesion given lack of association between *STAG2* mutations and complex karyotype and aneuploidy (10, 18). STAG1- versus STAG2-containing complexes have been recently shown to differentially contribute to chromatin organization, facilitating longer loops at topologically associating domain (TAD) boundaries and shorter, more transient nested enhancer-promoter contacts, respectively (19–22). Furthermore, deficiency of *Stag2* and RUNX family transcription factor 1 (*Runx1*) has been shown to disrupt enhancer-promoter looping and affect transcriptional pausing leading to selective gene dysregulation (22).

We sought to determine the effects of *STAG2* mutations observed in patients on the cohesin complex composition and genetic dependencies, with the goal of understanding the mechanisms by which these mutations contribute to cellular transformation and how cohesin-mutant malignancies could be therapeutically targeted.

## Results

### Genetic synthetic vulnerabilities in STAG2-mutant cells.

To study the cellular consequences of cohesin mutations in myeloid malignancies, we used CRISPR/Cas9 to engineer a spectrum of predicted LOF *STAG2*, *SMC3*, and *RAD21* mutations identified in patients in AML cell lines WT for all cohesin subunits and modulators. The different mutants largely phenocopy one another and are consistent with loss of function of STAG2 or haploinsufficiency of SMC3 or RAD21 (Supplemental Figure 1, A–G; supplemental material available online with this article; https://doi.org/10.1172/jci.insight.142149DS1; see Methods). We first focused on STAG2, the most frequently mutated subunit of the cohesin complex, and hypothesized that different *STAG2* mutations would be associated with mutant-specific genetic dependencies.

**Figure 1:**
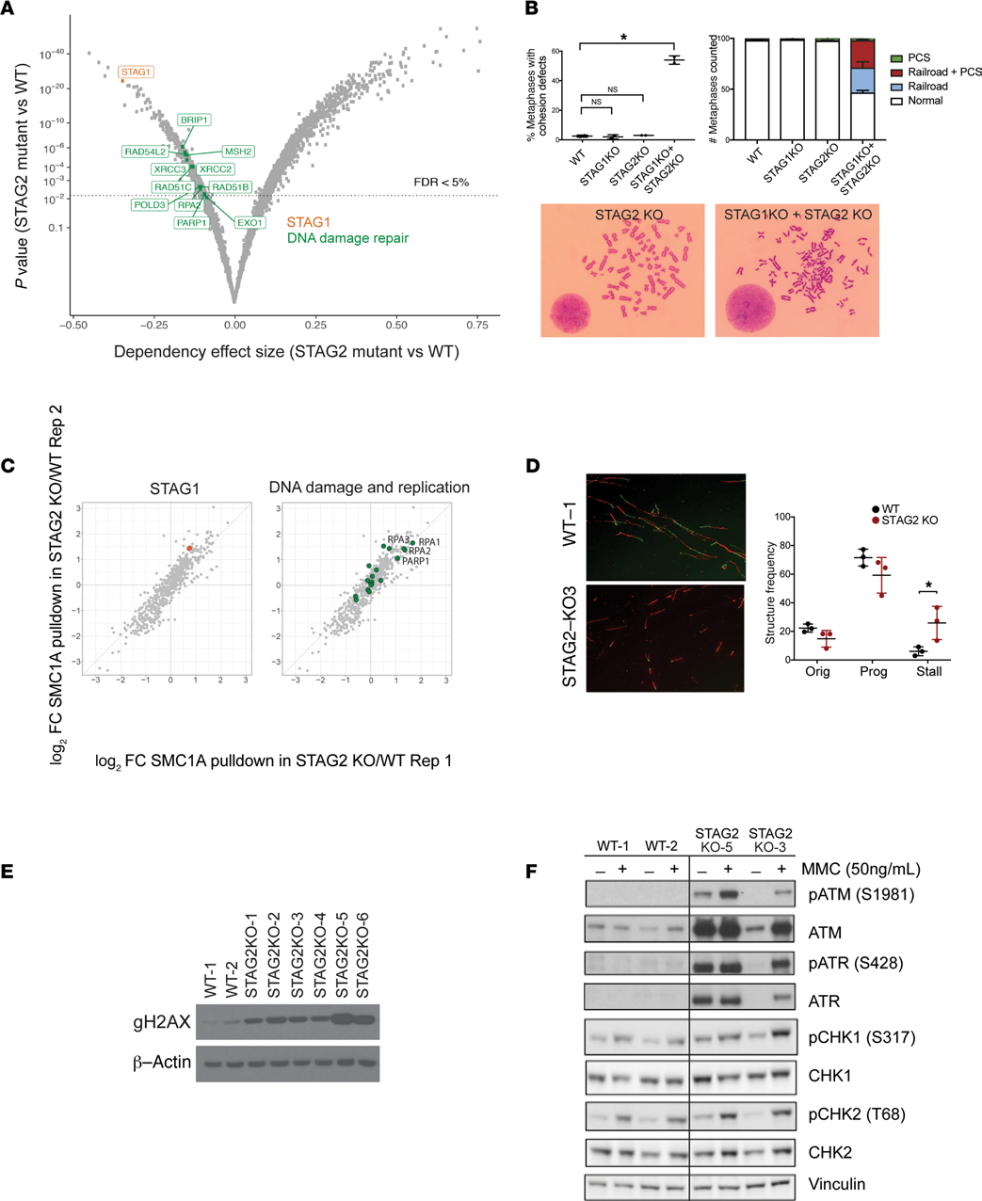
Identification of DNA replication and damage repair as a dependency in *STAG2*-mutant cells. (**A**) Volcano plot depicting differential dependencies in *STAG2*-mutant versus WT cells. Composite data for 5 *STAG2*-mutant cell lines (U937 STAG2-KO2, STAG2-KO3, KOC5, KOD5C, KOG8B) and 6 *STAG2*-WT cell lines (U937 WT-1, WT-2, NCB1, NCB12, NCB2A, NCC4) are shown. Respective sets of genes representing dependency in *STAG2*-mutant over WT cells with FDR < 5% are shown in color. (**B**) Cohesion defect analysis in WT, *STAG1*-, *STAG2*-, and double *STAG1*/*STAG2*–knockout cells. Mean ± SD is shown for 2 independent biological replicates of *STAG2*-WT (U937 WT-1, WT-2) and *STAG2*-knockout cells (U937 STAG2-KO3, STAG2-KO4) transduced with STAG1 or control sgRNAs. For each sample 100 metaphase spreads were scored. *P < 0.0001 (1-way ANOVA). PCS, premature centromere separation; Railroad, railroad chromosomes. (**C**) Log_2_ fold change (FC) of protein enrichment after SMC1A IP-MS in WT and *STAG2*-knockout (KO) cells. Rep1 and Rep2 correspond to different mutant clones. Proteins belonging to the DNA damage repair and replication gene set are highlighted in green. Enrichment *P* value was determined using a 1-tailed Fisher’s exact test (*P* = 0.024). U937 WT-1, WT-2, STAG2-KO5, and STAG2-KO6 were used in this experiment. (**D**) Representative images depicting replication structures of single combed DNA molecules labeled with IdU (red) and CIdU (green) in WT and *STAG2*-knockout cells. Quantification of replication origin firing (Orig), progressing replication forks (Prog), and stalled replication forks (Stall) in WT and *STAG2*-mutant cells. Data from 3 WT (U937 WT-1, WT-2, WT-3) and 3 *STAG2*-KO (U937 STAG2-KO2, STAG2-KO3, STAG2-KO4) cell lines combined. *P* < 0.05 (unpaired Student’s *t* test). Original magnification, 400×. (**E**) Western blotting for γ-H2Ax. U937 WT-1, WT-2, STAG2 KO-1, STAG2 KO-2, STAG2 KO-3, STAG2 KO-4, STAG2 KO-5, and STAG2 KO-6 were used. β-Actin was a loading control. (**F**) Western blotting for DNA damage checkpoint proteins ATM, phosphorylated ATM (p-ATM), ATR, p-ATR, CHK1, p-CHK1, CHK2, and p-CHK2 in WT and *STAG2*-mutant cells in the presence and absence of mitomycin C (MMC). Vinculin was a loading control.

We performed genome-scale CRISPR/Cas9 screens in 6 WT and 5 *STAG2*-mutant U937 cell lines representing different *STAG2* mutations using the Avana sgRNA library, which targets a total of 20,000 protein-coding genes with 4 unique sgRNAs per gene and includes 1000 nontargeting sgRNA controls (23). We observed that *STAG2*-mutant cells were strongly dependent on *STAG1*, as has been recently reported in the context of bladder cancer and Ewing sarcoma cell lines (24, 25) (Figure 1A and Supplemental Figure 2A). Since both STAG1- and STAG2-containing cohesin complexes participate in sister chromatid cohesion, we examined whether loss of *STAG1* in *STAG2*-deficient cells would lead to aberrant sister chromatid cohesion. We found that loss of both *STAG1* and *STAG2*, but not loss of either one alone, led to sister chromatid cohesion defects as assessed by premature centromere separation and railroad chromosomes (Figure 1B), providing a mechanistic basis for the synthetic lethality of *STAG1* and *STAG2*. The absence of sister chromatid cohesion defects in cells harboring loss of STAG2 alone is in agreement with lack of aneuploidy or complex karyotype in patients with *STAG2*-mutant MDS and AML (5, 10, 26), as well as previous studies in yeast suggesting that sister chromatid cohesion is unaffected with up to 87% loss of cohesin levels (27). Therefore, *STAG2*-mutant cells are dependent on the presence of STAG1 and do not have overt sister chromatid cohesion defects unless accompanied by a simultaneous loss of STAG1.

In addition to *STAG1*, we identified preferential dependency of *STAG2*-mutant cells on multiple components of the DNA damage repair and replication machinery (Figure 1A), as well as lineage-defining transcription factors, and genes involved in mRNA processing (Supplemental Table 1). Of note, we identified multiple members of the base excision repair [poly(ADP-ribose) polymerase 1; PARP1], homologous recombination (BRIP1, RAD51B, RAD51C, RAD54L2, XRCC2, XRCC3, PARP1), mismatch repair (MSH2; DNA polymerase delta 3, accessory subunit [POLD3]; EXO1), and DNA replication (replication protein A2 [RPA2], POLD3) machineries as specific synthetic vulnerabilities of *STAG2*-mutant cells (Figure 1A). Therefore, we observed differential dependency of *STAG2*-mutant cells on DNA damage repair and replication-associated pathways.

### Altered cohesin complex composition and interactome in STAG2-mutant cells.

Having demonstrated that *STAG2* mutations lead to genetic dependencies on STAG1 and DNA damage repair and replication, we hypothesized that these cohesin-dependent vulnerabilities could be associated with mutant-specific protein complex alterations. To examine the effect of *STAG2* loss on the composition of the cohesin complex, we employed immunoprecipitation using an antibody against the core cohesin ring subunit SMC1A followed by quantitative mass spectrometry (IP-MS) (Supplemental Figure 2B). In WT cells, we detected all members of the cohesin complex, including direct binding partners SMC3 and RAD21, as well as indirect binding partners STAG1, STAG2, and PDS5B (Supplemental Figure 2, C and D; and Supplemental Table 2A). In comparison, examination of *STAG2*-mutant clones revealed STAG1 to be among the most enriched proteins preferentially incorporated into the cohesin complex in cohesin-mutant cells (hereafter referred to as STAG1-cohesin complex for simplicity) (Figure 1C and Supplemental Table 2B). We validated this switch to STAG1-containing complexes in *STAG2*-mutant cells using immunoprecipitation followed by Western blotting (Supplemental Figure 2E). A switch from STAG2 to STAG1-cohesin complexes was consistent with the dependency of *STAG2*-mutant cells on STAG1 (Figure 1A). Unexpectedly, we also observed increased incorporation of STAG1 into cohesin complexes in cells with heterozygous mutation of a non-STAG1/2 paralog cohesin subunit, SMC3 (Supplemental Figure 2, F and G; and Supplemental Table 2C). These studies provide evidence that mutation of either *STAG2* or *SMC3* causes a shift to STAG1-containing cohesin complexes.

We next examined whether the shift from STAG2- to STAG1-containing cohesin complexes is associated with additional changes in its interactome that could explain the genetic dependencies that we had observed. We observed a significant increase in the interaction of the STAG1-cohesin complex with proteins involved in DNA replication and DNA damage repair (e.g., PARP1, RPA1–3; *P* = 0.024) (Figure 1C), transcription factors, and splicing proteins (Supplemental Table 2B). Similarly, we observed changes in the interaction of the STAG1-cohesin complex with the DNA damage repair, replication, and splicing machinery in *SMC3*-mutant cells (Supplemental Figure 2F), suggesting that mutations affecting different cohesin subunits may affect the cohesin complex structure and interactome concordantly. These findings demonstrate a high concordance between the cellular processes highlighted by IP-MS experiments and genetic dependency screens in cohesin-mutant cells, especially as it relates to DNA replication and damage repair.

### Stalled replication forks and accumulation of dsDNA breaks in STAG2-mutant cells.

Having found that *STAG2* mutations were associated with a genetic dependency on components of the DNA replication and damage repair pathways, and altered interaction of these proteins with the cohesin complex, we examined whether cells bearing *STAG2* mutations accumulate DNA damage. Cohesin has been shown to organize chromatin loops at DNA replication factories in order to mediate replication stress tolerance and restart stalled replication forks (28–32). Since aberrant replication forks could serve as a potential source of DNA damage in cohesin-mutant cells, we investigated DNA replication fork processivity using a DNA fiber assay (33). Loss of STAG2 was associated with an increase in the number of stalled replication forks (6% in WT cells versus 25% in *STAG2*-mutant cells, *P* < 0.05) and a tendency to lose replication origin firing (Figure 1D). Furthermore, we observed significant replication fork asymmetry and a 27% decrease in the replication fork rate in *STAG2*-mutant cells (Supplemental Figure 3, A and B), both of which are consistent with replication fork slowing and stalling. Therefore, *STAG2* mutations are associated with abnormal replication fork processivity and stalling, a phenomenon that may be due to aberrant spatial arrangement of replication origins and/or ineffective restarting of replication forks in the absence of a normal cohesin complex.

Stalled replication forks can lead to DNA damage, including dsDNA breaks, if inappropriately resolved. The cohesin complex has been previously implicated in dsDNA break repair and intra-S and G2M checkpoint (34). STAG1- versus STAG2-containing cohesin complexes have been shown to affect repair pathway choice, with STAG2-cohesin complex being preferentially involved in sister chromatid homologous recombination repair (35). We therefore examined γ-H2Ax accumulation using immunoblotting as an indicator of dsDNA breaks. We observed accumulation of γ-H2Ax staining under homeostatic conditions across all *STAG2*-mutant cell lines (Figure 1E). This was associated with activation of the ataxia telangiectasia mutated (ATM) and ataxia telangiectasia mutated and Rad3 related (ATR) DNA damage checkpoints as assessed by phosphorylation of ATM and ATR proteins (Figure 1F), even in the absence of mitomycin C treatment, which is predicted to induce DNA damage by blocking both replication and transcription. These data therefore indicate that one of the functional consequences of STAG1-cohesin complexes is aberrant DNA damage repair and increased genomic instability.

### STAG2-mutant cells are sensitive to PARP inhibition in vitro and in vivo.

**Figure 2:**
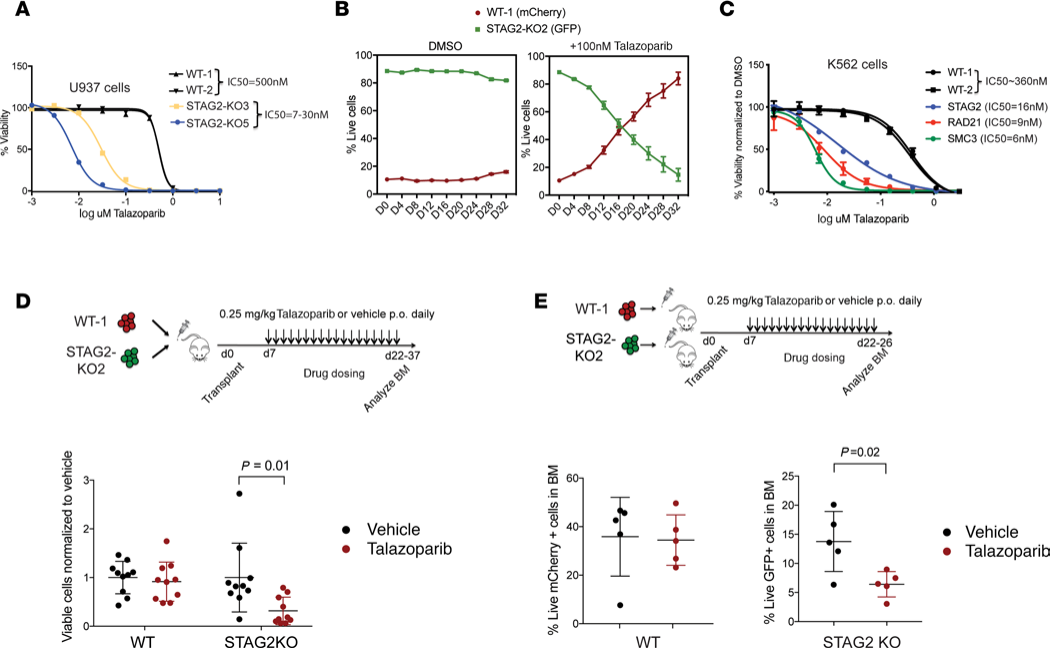
*STAG2-*mutant AML cell lines are more sensitive to PARP inhibition in vitro and in vivo. (**A**) Drug dose response curves of WT and *STAG2*-mutant U937 cell lines treated with the PARP inhibitor talazoparib. U937 WT-1, WT-2, STAG2 KO-3, and STAG2 KO-5 cells were used for this experiment. IC_50_ was calculated on day 12 of treatment. Error bars represent SD of measurements of triplicate technical replicates. (**B**) Competition assay with WT (U937 WT-1-mCherry) and *STAG2*-knockout (U937 STAG2-KO2-GFP) cells mixed in 1:10 ratio in the presence of DMSO or talazoparib (100 nM) in vitro. Error bars represent SD of measurements of triplicate technical replicates. (**C**) Drug dose response curves of WT and *STAG2*-, *SMC3*-, and *RAD21*-mutant K562 clones treated with talazoparib. IC_50_ was calculated on day 12 of treatment. Error bars represent SD of measurements of 3 technical replicates. (**D**) Schematic of the in vivo drug treatment of WT and *STAG2*KO xenografts. WT (U937 WT-1-mCherry) and *STAG2*-knockout (U937 STAG2-KO2-GFP) cells were mixed 1:1 and transplanted into NOD/SCID IL-2Rγ null Tg(IL3, CSF2, KITL) (NSGS) recipients and dosed with talazoparib or vehicle by oral gavage at 0.25 mg/kg once a day. Percentage of live GFP^+^ or mCherry^+^ cells in the bone marrow was determined using flow cytometry. Mean ± SD is shown. *P* = 0.01 (Student’s *t* test). *n* = 10 mice per group. (**E**) Schematic of the in vivo drug treatment of WT and *STAG2*-knockout xenografts. WT (U937 WT-1 mCherry) or *STAG2*-knockout (U937 STAG2-KO2-GFP) cells were transplanted into NSGS recipients and dosed with talazoparib or vehicle by oral gavage at 0.25 mg/kg once a day. Percentage of live GFP^+^ or mCherry^+^ cells was determined using flow cytometry. Mean ± SD is shown. *P* = 0.02 (Student’s *t* test). *n* = 5 mice per group.

We next addressed whether altered DNA damage response in *STAG2*-mutant cells creates a vulnerability that can be exploited therapeutically. In our CRISPR screen for genetic dependencies, we found *STAG2*-mutant cells to have a genetic dependency on *PARP1* (Figure 1A, P** = 0.006), and in our IP-MS experiments, we found an increased association of PARP1 with the cohesin complex in *STAG2*-mutant cells (Figure 1C, log_2_FC = 1.03). Furthermore, genetic screens in *Saccharomyces cerevisiae* and *Caenorhabditis* *elegans* previously identified synthetic lethality interactions between replication fork mediators, including PARP genes, and mutant cohesin (36). We therefore tested sensitivity of *STAG2*-mutant cells to PARP inhibition. PARP inhibitors, including talazoparib, inhibit PARP catalytic activity and trap PARP at the sites of DNA damage, rendering cells that are dependent on nonhomologous end joining repair of dsDNA breaks particularly sensitive to these agents. Treatment with talazoparib resulted in approximately 70-fold increased sensitivity of *STAG2*-mutant cells as compared with WT cells (Figure 2A). In addition, *STAG2*-mutant cells were outcompeted by WT cells in the presence of talazoparib (Figure 2B). We also sought to determine whether mutations in other components of the cohesin complex result in a similar dependency on PARP inhibition. We found that heterozygous inactivation of *SMC3* or *RAD21* was associated with increased sensitivity to PARP inhibition to a similar extent as *STAG2* mutations in U937 and K562 cells (Figure 2C and Supplemental Figure 3C), suggesting that all core cohesin complex mutations we have tested may act as biomarkers of response to PARP inhibition.

We reproduced this effect in xenograft animals injected with individual or competitive mixtures of WT and *STAG2*-mutant AML cells and observed a genotype-specific effect of talazoparib on *STAG2*-mutant cells (Figure 2, D and E). Finally, in order to examine whether response to PARP inhibition in primary human leukemia cells is *STAG2* mutation dependent, we treated *STAG2*-mutant and WT primary AML patient samples with talazoparib and noted a dose- and genotype-dependent sensitivity to the drug (Supplemental Figure 3D). Cumulatively, these studies indicate that cohesin complex mutations result in increased association of DNA repair factors with mutant STAG1-containing cohesin complexes, impaired DNA damage repair, and increased sensitivity to PARP inhibitors.

### Development of cohesin-mutant mouse models of MDS and AML.

**Figure 3:**
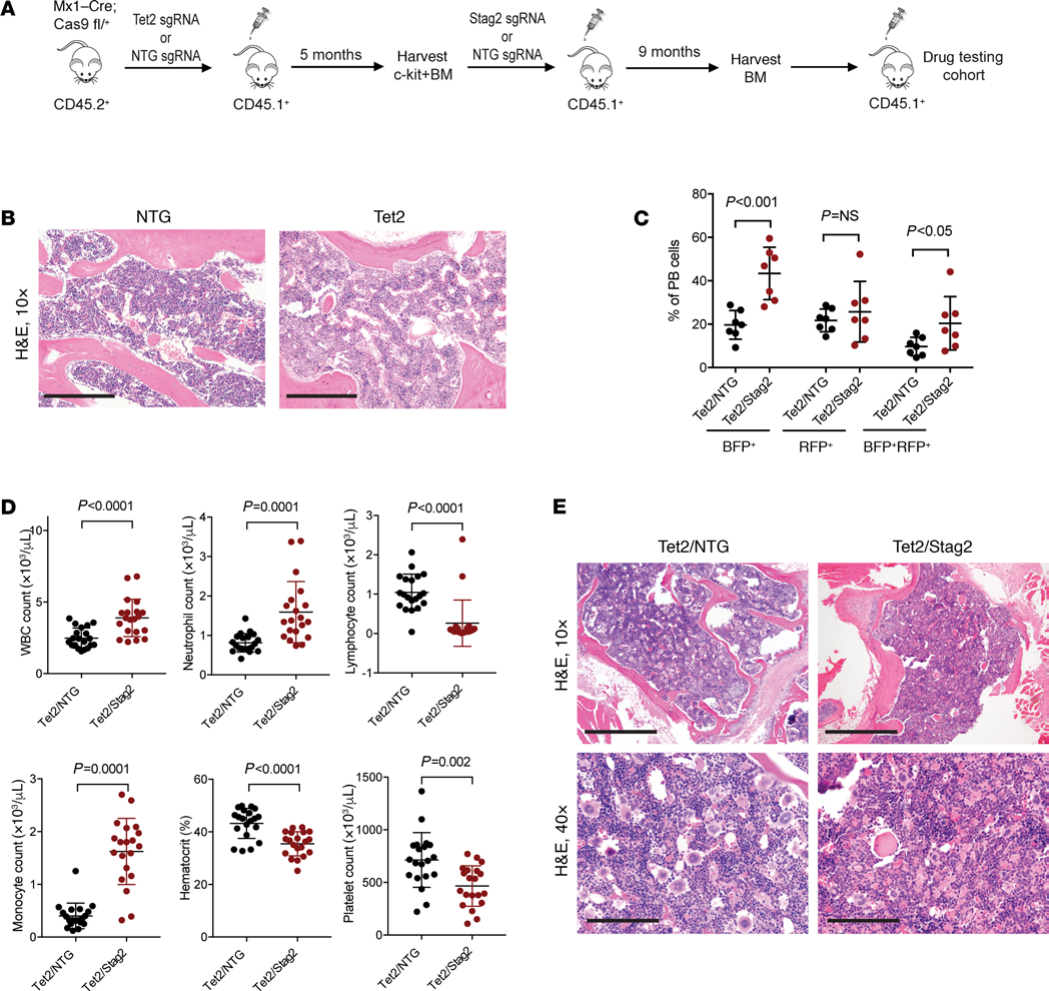
Development of primary models of cohesin-mutant MDS. (**A**) Schematic of the sequential bone marrow transplant used to generate *Tet2/Stag2*-mutant models of myeloid disease. (**B**) Morphologic evaluation of bone marrow section of mice injected with NTG and *Tet2*-mutant cells. H&E staining, 10× magnification. No appreciable differences were observed. Scale bar: 0.5 mm. (**C**) Flow cytometry analysis of peripheral blood (PB) samples of mice sequentially transplanted with *Tet2/*NTG and *Tet2/Stag2* 3 months after transplantation. Blue fluorescent protein (BFP) reporter is linked to expression of sgRNA targeting *Stag2*, and red fluorescent protein (RFP) reporter is linked to expression of sgRNA targeting *Tet2*. Expansion of BFP^+^ and BFP^+^RFP^+^ cells in *Tet2/Stag2* animals. *n* = 7 per arm. Mean ± SD shown. (**D**) Absolute white blood cell (WBC) count, neutrophil count, lymphocyte count, monocyte count, hematocrit, and platelet count were measured in *Tet2/*NTG and *Tet2/Stag2*-mutant mice 12 weeks after bone marrow transplantation. Mean ± SD is shown. *P* values were determined using the Student’s *t* test. *n* = 20 mice per group. (**E**) Morphologic evaluation of bone marrow of a representative *Tet2/*NTG and *Tet2/Stag2*-mutant mouse shows a decrease in megakaryocytes and increased erythrophagocytosis in *Tet2/Stag2*-mutant mice. Images were stained using H&E and imaged at 10× (scale bar: 0.5 mm) and 40× (scale bar: 0.125 mm) original magnification.

In order to extend our observations made in AML cell lines to primary models of *STAG2*-mutant myeloid disease, we developed a syngeneic mouse model in which *Stag2* mutations arise as secondary lesions in the background of clonal hematopoiesis driven by tet methylcytosine dioxygenase 2 (*Tet2*) mutations, as is seen in the development of human MDS (4) (Figure 3A). HSPCs (Lineage^–^Sca1^+^c-Kit^+^ cells) harvested from Mx1-Cre *Cas9* heterozygous C57BL/6 mice were transduced with sgRNA targeting *Tet2* or nontargeting sgRNA (NTG) and transplanted into lethally irradiated SJL recipient mice. Engraftment and clonal expansion of *Tet2*-mutant cells were confirmed by fluorescent protein reporter expression and next-generation sequencing (37) and were not associated with an overt phenotype (Figure 3B). Next, c-Kit–enriched bone marrow cells from mice with *Tet2* mutations (*Tet2* indel fraction 0.62) were transduced with sgRNAs targeting *Stag2* or NTG and transplanted into secondary recipient mice. Cells with *Tet2/Stag2* genetic editing expanded relative to *Tet2/*NTG cells by 2 months posttransplantation (Figure 3C). In contrast to *Tet2*-only mutant mice, *Tet2/Stag2*-mutant mice developed leukocytosis, absolute monocytosis, anemia, and thrombocytopenia (Figure 3D).

Morphologic evaluation of *Tet2/Stag2* bone marrow revealed fewer megakaryocytes and increased hemophagocytosis consistent with macrophage activation in comparison with *Tet2*-only mutant mice (Figure 3E). Next-generation sequencing (NGS) confirmed predicted LOF frameshift mutations in *Tet2* and *Stag2* (mean *Tet2* indel fraction 0.80 in *Tet2/*NTG mice; mean *Tet2* and *Stag2* indel fractions 0.64 and 0.63, respectively, in *Tet2/Stag2* mice). In concordance with our AML cell line data, *Tet2/Stag2*-mutant bone marrow cells exhibited higher levels of dsDNA breaks and increased sensitivity to treatment with talazoparib when cultured in vitro (Supplemental Figure 3, E–G). Ex vivo low-dose irradiation of bone marrow cells led to an increase in dsDNA breaks in *Tet2*/NTG cells treated with vehicle but did not appreciably increase already elevated levels of dsDNA breaks in *Tet2/Stag2* or any of the talazoparib-treated cells. These studies demonstrate that our CRISPR/Cas9 model with sequential acquisition of *Tet2* and *Stag2* mutations results in aberrant hematopoiesis with *Stag2*-mutant–specific alterations in DNA damage response.

### Talazoparib depletes cohesin-mutant clones in in vivo models of MDS and AML.

**Figure 4:**
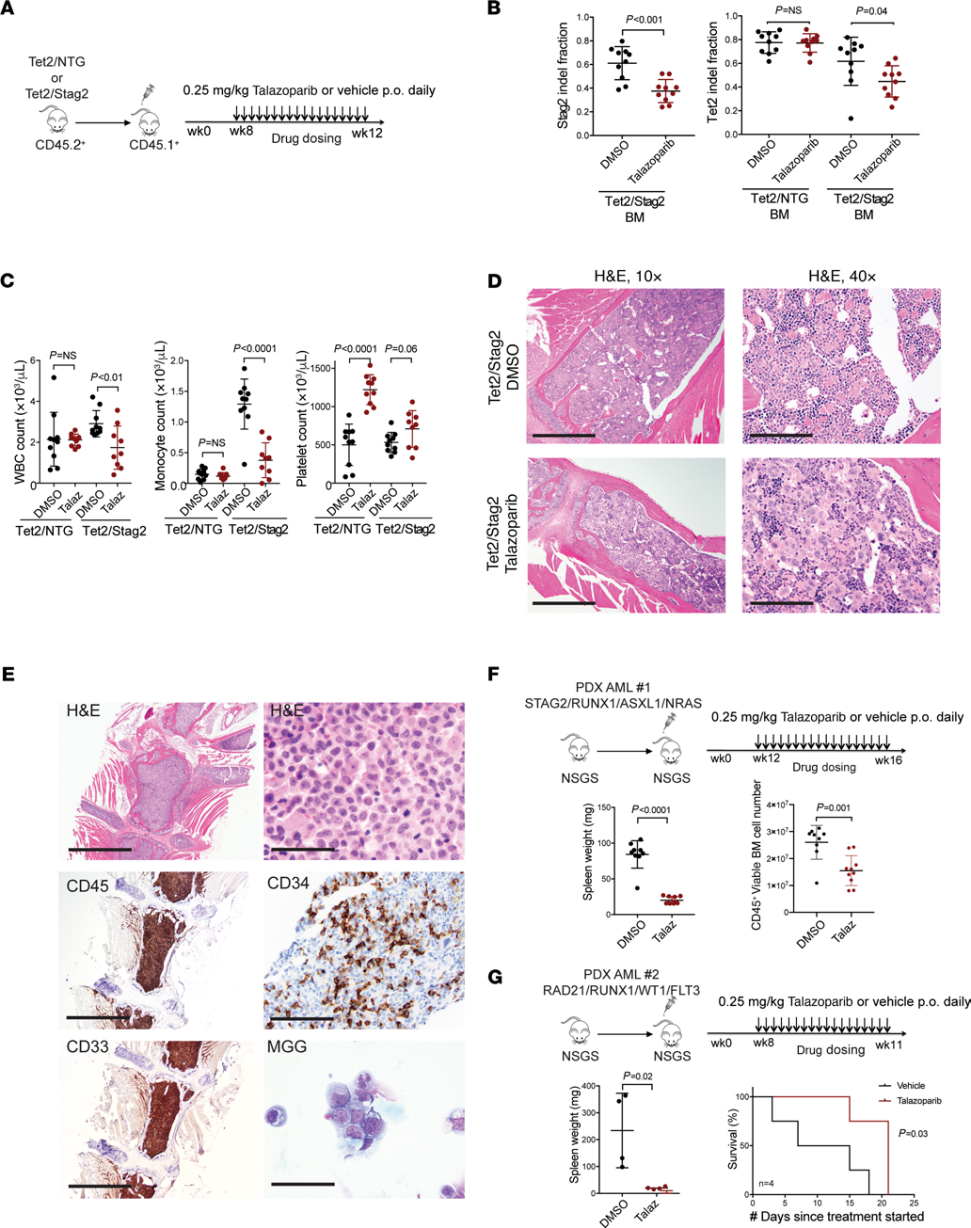
Talazoparib treatment preferentially depletes cohesin-mutant clones in primary mouse and human cell in vivo models of cohesin-mutant myeloid diseases. (**A**) Schematic of the in vivo drug treatment of *Tet2/*NTG and *Tet2/Stag2*-mutant mice with talazoparib. (**B**) Bone marrow analysis of *Stag2* and *Tet2* indel fraction by NGS demonstrates a genotype-specific response to talazoparib treatment in *Tet2/Stag2* but not *Tet2/*NTG-mutant clones. Mean ± SD is shown. *P* values were determined using Student’s *t* test. *n* = 10 mice/group. (**C**) Complete blood count analysis shows normalization of the WBC, monocyte, and platelet counts in talazoparib-treated *Tet2/Stag2* animals. Mean ± SD is shown. *P* values were determined using Student’s *t* test. *n* = 10 mice/group. (**D**) Morphologic evaluation of bone marrow of representative *Tet2/Stag2*-mutant mice treated with talazoparib or DMSO shows an increased megakaryocyte number and persistent erythrophagocytosis in *Tet2/Stag2*-mutant mice treated with talazoparib. Images were stained using H&E and imaged at 10× (scale bar: 0.5 mm) and 40× (scale bar: 0.125 mm) original magnification. (**E**) Generation of a *STAG2*-mutant AML PDX model in NSGS mice. Staining with H&E, modified Giemsa May-Grünwald (MGG), and immunohistochemistry shows expansion of immature CD45^+^CD34^+^CD33^+^ myeloid blasts in the bone marrow. Images were taken using 10× (scale bar: 0.5 mm) and 100× original magnification (scale bar: 0.05 mm). (**F**) Schematic of the in vivo drug treatment of *STAG2*-mutant AML PDX model. Mice howed a decrease in spleen size and human CD45^+^ bone marrow disease burden in the talazoparib-treated arm. Mean ± SD is shown. *P* values were determined using Student’s *t* test. *n* = 9–10 mice/group. (**G**) Schematic of the in vivo drug treatment of *RAD21*-mutant AML PDX model. Treatment with talazoparib led to a decrease in spleen size and improved overall survival. Mean ± SD is shown. *P* values were determined using Student’s *t* test and Kaplan-Meier survival analysis. *n* = 4 mice/group.

*Tet2/Stag2* and*Tet2*-mutant clones and the associated hematologic phenotypes were serially transplantable, enabling evaluation of genotype-specific response to the PARP1 inhibitor talazoparib in vivo. Forty recipient mice transplanted with *Tet2* or *Tet2/Stag2* mutant bone marrow cells were stratified into treatment groups with talazoparib or vehicle (Figure 4A). Expression of congenic markers and fluorescent reporters linked to *Tet2* and *Stag2* sgRNA expression were used to monitor mice during 4 weeks of treatment. *Tet2/Stag2*-mutant mice but not *Tet2*-only mutant mice treated with talazoparib demonstrated a significant loss of mutant cells as determined by NGS and flow cytometry (Figure 4B and Supplemental Figure 4A). In addition, we observed normalization of leukocytosis, monocytosis, and thrombocytopenia in *Tet2/Stag2*-mutant mice treated with talazoparib (Figure 4C), which was associated with increased numbers of megakaryocytes on blinded review (Figure 4D and Supplemental Figure 4B, P** = 0.007).

We next wanted to examine whether response to PARP inhibition is cohesin mutation dependent in primary patient-derived leukemia cell xenografts. We developed 2 unique serially transplantable patient-derived xenograft (PDX) models of *STAG2*- and *RAD21*-mutant AML (Figure 4E and Supplemental Figure 4, C and E) and evaluated the efficacy of talazoparib in both models in vivo. We noted a decrease in disease burden and increased survival of cohesin-mutant PDX models treated with talazoparib as compared with vehicle (Figure 4, F and G; and Supplemental Figure 4, D and F). Therefore, in both primary mouse HSPCs and human AML cells, *STAG2*-mutant cells are selectively sensitive to treatment with talazoparib.

### STAG2 loss alters chromatin compartmentalization and looping.

**Figure 5:**
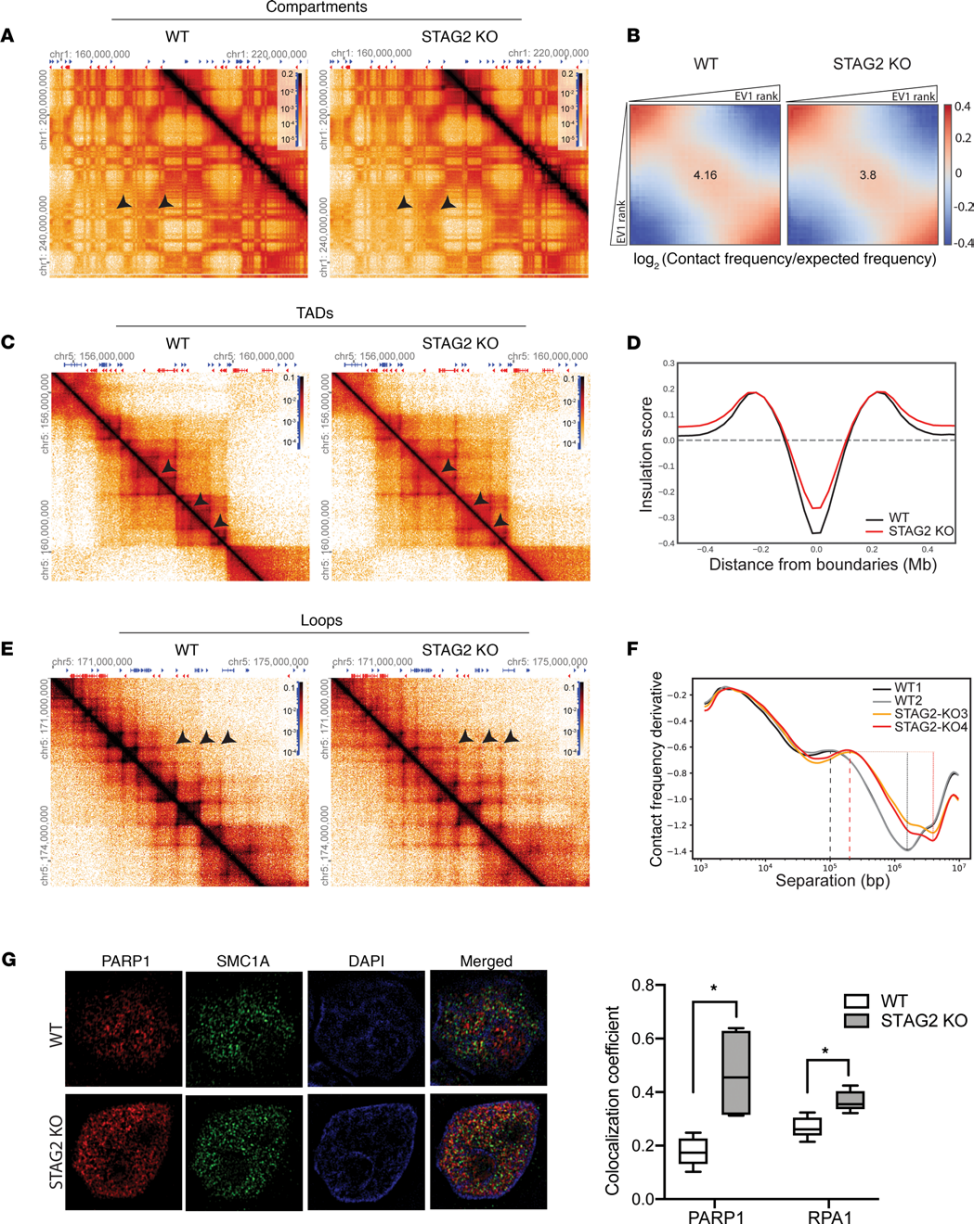
STAG2 loss disrupts normal chromatin folding and association with DNA replication and damage repair proteins. (**A**) Hi-C interaction maps for chromosome 1 binned at 100 kb in WT vs. *STAG2*-knockout cells to visualize compartments. Arrowheads depict examples of weakened compartments. (**B**) Saddle plots of Hi-C data binned at 100 kb resolution normalized by genomic distance. The saddle plot demonstrates global weakening of compartments in STAG2-knockout cells. Heatmaps were generated using Hi-glass from pooled reads from 2 independent WT (U937 WT-1, WT-2) and *STAG2*-knockout (U937 STAG2-KO3, STAG2-KO4) cell lines. EV1, first eigenvector. (**C**) Hi-C interaction maps for a genomic region in chromosome 5 binned at 25 kb in WT vs. *STAG2*-knockout cells to visualize TADs. Heatmaps were generated using Hi-glass from pooled reads from 2 independent WT (U937 WT-1, WT-2) and *STAG2*-knockout (U937 STAG2-KO3, STAG2-KO4) cell lines. Arrowheads depict examples of loss of TAD insulation. (**D**) Insulation score (42) as a function of distance from TAD boundaries demonstrates global weakening of insulation at TAD boundaries. (**E**) Hi-C interaction maps for a genomic region in chromosome 5 binned at 10 kb in WT vs. *STAG2*-knockout cells to visualize loops. Arrowheads depict examples of gain of longer loops. (**F**) Relationship between interaction frequency (*P*) and genomic distance (*s*) to estimate the average loop size and density demonstrates longer extruded loops in *STAG2*-knockout cells compared with WT cells (represented by dashed lines, U937 WT1, WT2 ~100 kb; U937 STAG2-KO3, KO4 ~200 kb) and the density of loops is reduced in *STAG2*-knockout cells (represented by dotted lines). (**G**) Structured illumination microscopy of SMC1A and PARP1 in WT (U937 WT-1, WT-2) and *STAG2*-knockout (U937 STAG2-KO-3, STAG2-KO5) cells. Fluorescence signal displayed alone and merged with the nuclear Hoechst stain. Quantification of colocalization of SMC1A with PARP1 or RPA1 was determined using Manders colocalization coefficient. Original magnification, 100×. Box and whiskers represent mean ± Tukey’s. *P < 0.0001, unpaired Student’s *t* test.

In order to understand the impact of the aberrant cohesin complex on chromatin compartmentalization and looping, which have been previously linked to DNA replication stress and damage (38), we performed the chromosome conformation analysis Hi-C (39, 40) in a set of *STAG2*-WT and -knockout AML cell lines. This unbiased genome-wide chromosome conformation analysis enables evaluation of chromatin organization at multiple tiers of genome organization, including compartments, TADs, and loops. Compartments are apparent by the plaid pattern of interaction in Hi-C interaction maps (Figure 5A). Analysis of this pattern is routinely performed by principal component analysis, where principal component 1 (PC1) typically captures the positions of compartment domains. The strength of compartmentalization can then be visualized and quantified by rearranging chromatin interaction maps by ordering loci according to their PC1 value to produce compartmentalization “saddle plots” (41). We observed a global weakening of compartmentalization and spatial segregation of active and inactive chromatin domains in *STAG2*-knockout cells (Figure 5B), consistent with more intermixing between expression-rich “A compartments” and expression-poor “B compartments” as defined previously (39). Also, the number and location of TAD boundaries, determined by insulation score analysis (42), was largely preserved between *STAG2*-WT and -mutant cells (Supplemental Figure 5A), but the strength of TAD boundary insulation was globally weakened (Figure 5, C and D; and Supplemental Figure 5B).

Finally, we assessed the effects of STAG2 loss on the strength and size distribution of positioned loops that are apparent as dots in Hi-C interaction maps and correspond to enriched CTCF-CTCF (CCCTC binding factor–CCCTC binding factor) interactions at the bases of the loops (43). Visual inspection of the heatmaps revealed stronger dots farther away from the diagonal (arrows in Figure 5E). Genome-wide average loop size and loop density can be estimated by analysis of the relationship between interaction frequency (*P*) and genomic distance (*s*) (Supplemental Figure 5C). Specifically, the position of a local maximum in the derivative of *P*(*s*) has been previously shown to represent the average loop size (44). In *STAG2*-WT cells loops were on average 100–200 kb, while in *STAG2*-knockout cells the average loop size was 200–300 kb. In addition, we noted the loop density to be reduced in *STAG2*-knockout cells (Figure 5F).

Combined, these findings show that in the absence of STAG2, STAG1-containing cohesin complexes extrude larger and somewhat fewer loops. Possibly, STAG1-containing cohesin complexes are blocked less efficiently at CTCF sites (resulting in reduced insulation at CTCF sites and TAD boundaries), allowing the loops to more frequently pass CTCF sites and the formation of larger loops. This longer range extrusion process is also expected to lead to more intermixing of A and B compartments.

### STAG2 loss leads to increased colocalization of cohesin with DNA replication and damage repair proteins.

A unifying hypothesis for genetic and pharmacologic dependencies on DNA replication and damage response is that altered chromatin insulation and compartment structure in cohesin-mutant cells leads to shifts in the physical colocalization of proteins involved in these cellular processes. We addressed this hypothesis using super-resolution microscopy. Staining for SMC1A protein, we identified WT and mutant cohesin protein complex aggregates as distinct nuclear puncta (Figure 5G). We observed increased colocalization of the STAG1-cohesin complex with PARP1 and RPA1 (Figure 5G). Therefore, we observed *STAG2* mutant–dependent alterations in the physical colocalization of the cohesin complex with DNA damage, concordant with our previously identified changes in the cohesin interactome and genetic dependencies. Put in context with our Hi-C analysis, we hypothesize that these changes may be driven by increased processivity of the STAG1-cohesin complex associated with a loss of TAD boundary insulation and longer loop extrusion.

These findings are consistent with a model in which cohesin complexes in WT and cohesin-mutant cells, defined by their unique composition, have differential ability to maintain chromatin organization as it relates to spatial organization of DNA damage repair machinery. Decreased colocalization and function of these components in turn creates vulnerabilities that have the potential to be exploited therapeutically in patients with cohesin-mutated malignancies.

## Discussion

Our studies establish a role for DNA damage, DNA replication, and chromatin architecture in the biology and therapeutic targeting of cohesin-mutant myeloid malignancies. Using genetic screens, IP-MS, chromatin conformation studies, and super-resolution microscopy, we demonstrate that the introduction of cohesin mutations results in a switch from STAG2 to STAG1-cohesin complexes and differential cohesin dependence on DNA damage repair and replication. We observed global spatial chromatin reorganization, including longer loop extrusion, loss of insulation at TAD boundaries, and intermixing of compartments, associated with changes in cohesin interaction with DNA replication and damage machinery, which may explain the basis for the genetic dependency we observed. We extended these findings to LOF mutations in other members of the cohesin complex, including *SMC3* and *RAD21*, and identify cohesin mutations as potential biomarkers of response to treatment with PARP inhibitors.

There are currently very limited therapeutic options for patients with MDS, and no therapies have been identified with selective activity in cohesin-mutant disease. We found that cohesin mutations cause a 70-fold increased sensitivity to PARP inhibition and are a potential biomarker of PARP inhibitor sensitivity in cohesin-mutant myeloid malignancies. PARP inhibitors are currently approved by the FDA for treatment of breast and ovarian cancer in the context of germline *BRCA* mutations and have been previously tested in a phase I study of unselected patients with advanced hematologic malignancies (45). The effect of talazoparib monotherapy in cohesin-mutated AML or MDS with excess blasts is under investigation in a pilot proof-of-concept study (ClinicalTrials.gov identifier NCT03974217). *STAG2*-mutant glioblastoma cells have been previously shown to be sensitive to PARP inhibition in vitro (31, 46), and cohesin mutations may be potential biomarkers of PARP sensitivity in bladder cancer and Ewing sarcoma, where these mutations are common. In addition, combination treatment of hypomethylating agents and PARP inhibition should be considered given sensitivity of cohesin-mutant MDS and genetically engineered CD34^+^ cells to hypomethylating agents (9, 47), as well as increased sensitivity of PARP inhibition when administered with low-dose hypomethylating agents in preclinical studies (48). Finally, the role of PARP inhibition as a therapeutic strategy may be efficacious in a wider range of myeloid malignancies characterized by DNA damage repair defects, including *IDH1/2*-AML, *FLT3-ITD*-AML, splicing factor mutant AML, and AML1-ETO rearranged AML (49).

A number of studies have previously examined the effects of complete cohesin loss on chromatin organization, both in the context of an inducible loss of the essential cohesin subunit RAD21 in human cells and loss of the cohesin-loading factor Nipbl in mice (50, 51). These studies confirmed the essential function of the cohesin complex in the formation of topologically associated domains, which is independent of compartment organization. The models used to establish these findings represent a complete loss of cohesin complex, which is not tolerated in human cells and as a result is not implicated in human disease. Patients with cohesin-mutant myeloid malignancies never present with complete, biallelic inactivation of any cohesin subunit, with the notable exception of STAG2, which has a paralog, STAG1. Complete loss of STAG1 and STAG2 was synthetically lethal in our studies as well as in other recent reports (24, 25) and demonstrated in primary mouse HSPCs using *Stag2*-conditional knockout mice (15). The models used in our study aimed to recapitulate the extent of cohesin dysfunction that would be observed in human disease, using both engineered cell lines as well as primary mouse models of cohesin-mutant MDS and AML. Cohesin mutations are early but usually not initiating lesions in myeloid malignancies. Our in vivo model of cohesin-mutant MDS arising in a setting of *Tet2*-mutant clones recapitulates the sequential acquisition of cohesin mutations in the context of *Tet2*-mutant clonal hematopoiesis and the phenotype observed in patients. The approach we developed is highly adaptable and can be used to not only model different stages of disease progression in hematopoietic malignancies but also address the question of order of mutation acquisition and questions of necessity versus sufficiency for transformation using different genetic combinations.

Cohesin has been previously shown to organize chromatin loops at DNA replication factories into rosette-like structures, which allows for organized firing of multiple origins of replication and is disrupted with lower levels of cohesin leading to longer loop formation, similar to our observations with STAG2- versus STAG1-cohesin complexes (38). How exactly changes in chromatin organization mediated by the switch from STAG2- to STAG1-containing cohesin complexes in cohesin-mutant cancer cells lead to DNA replication stress and DNA damage repair defects in cells remain to be elucidated.

Significant progress has been made in the last 2 decades in our understanding of the spatial organization of eukaryotic genomes (reviewed in ref. 52). Acquisition of genetic lesions that affect chromatin architecture are common mechanisms of cellular transformation (53, 54). Our study sheds light on how mutations affecting the cohesin complex alter the biology of mutant MDS and leukemia cells in a manner that creates a specific therapeutic vulnerability to PARP inhibitors.

## Methods

### Cell lines.

U937 and K562 cells were obtained from the Broad Institute Cancer Cell Line Encyclopedia, where they were authenticated by short tandem repeat (original source of U937 and K562 cells was ATCC). Cells were grown in RPMI (Invitrogen, Thermo Fisher Scientific) and supplemented with 10% fetal calf serum (MilliporeSigma) and 100 U/mL penicillin and 100 μg/mL streptomycin (Invitrogen, Thermo Fisher Scientific).

### Mice.

Female 8- to 10-week-old NSGS mice (NOD/SCID; IL-2Rγ null; Tg[IL3, CSF2, KITL]; strain 013062) and female 8- to 10-week-old SJL mice (B6.SJL-Ptprc; strain 002014) were obtained from The Jackson Laboratory. Conditional Cas9-knockin mice (strain 026175) and Mx1-Cre mice (strain 003556), both obtained from The Jackson Laboratory, were bred to generate donors for bone marrow transplantation studies. All mice were housed in a pathogen-free animal facility in microisolator cages, and experiments were conducted according to an IACUC-approved protocol at the Broad Institute.

### Generation of cohesin-mutant single-cell clones.

U937-Cas9– and K562-Cas9–expressing cells were first generated by lentiviral transduction with a lentiviral vector pLX-311Cas9 (Addgene 96924). sgRNAs targeting *STAG2*, *SMC3*, and *RAD21* or NTGs were cloned into a minimal backbone plasmid (Addgene 41824) and transfected into U937-Cas9 or K562-Cas9 cells using nucleofection (Lonza, Nucleofector II). GFP^+^ cells were single cell sorted into 96-well plates and grown up into single-cell clones, which were confirmed by DNA sequencing of the targeted locus, as well as Western blotting. Of note, since *STAG2* is an X-linked gene that undergoes normal X inactivation in females, patients with predicted LOF *STAG2* mutations are predicted to lack normal STAG2 expression. Therefore, in both cases of U937 cells and K562 cells, which both carry 2 copies of the X chromosome, we screened for presence of homozygous *STAG2* LOF mutations. See Supplemental Methods.

The following sgRNAs targeting human genes were used in this study individually: STAG2 (exon 4): TCTGGTCCAAACCGAATGAA; STAG2 (A1): AATGTCTTACTGCTCTACAA; STAG2 (A2): CTGAATGTCATCCTCCCGT; STAG1 (G1): GGAATTAGAGGAGCAGGCCG; SMC3 (gRNA 1): GATAAAATGAGACGAGCCC; SMC3 (gRNA 5): GAATATACCATTTACAATC; RAD21 (gRNA 2): TGTGTTCGAGTGTAATTTAG; non-targeting (GC1): GACGGAGGCTAAGCGTCGCAA; non-targeting (GC3): GATCGTTTCCGCTTAACGGCG.

The single cell–derived cell lines generated and used in the paper are listed in Supplemental Table 3.

### CRISPR lentiviral transduction.

sgRNAs targeting STAG2 or SMC3 were cloned into lentiCRISPRv2 (Addgene 52961).

### Genome-wide CRISPR screening and differential dependency analysis.

A total of 5 *STAG2*-mutant and 6 *STAG2*-WT U937 cells expressing Cas9 were infected with the genome-wide human Avana LentiGuide-Puro CRISPR library (Broad Genetic Perturbations Platform), which contains approximately 75,000 sgRNAs targeting approximately 19,000 genes and 1000 controls, in 2 separate experiments, as previously described (23). See Supplemental Methods. Raw and processed data have been deposited at figshare: https://figshare.com/articles/dataset/CRISPR_screen_of_isogenic_STAG2_mutant_cell_lines/7120796

### Western blotting, immunoprecipitation, and mass spectrometry.

Details of Western blotting, immunoprecipitation, and IP-MS performed using iTRAQ and TMT6 labeling are detailed in the Supplemental Methods. See complete unedited blots in the supplemental material. The original mass spectra may be downloaded from Mass Spectrometry Interactive Virtual Environment (http://massive.ucsd.edu) using the identifier MSV000082970.

### In vitro talazoparib treatment and competition assays.

Talazoparib was purchased from Selleck Chemicals (S7048) and dissolved in DMSO. All drug dose response assays were conducted using CellTiter-Glo luminescent cell viability assay (Promega). Competition experiments were carried out with GFP- and mCherry-labeled cohesin-mutant cells mixed at different ratios and monitored by flow cytometry. See Supplemental Methods.

### DNA fiber assay, cohesion defect analysis, and super-resolution microscopy.

See Supplemental Methods.

### Bone marrow transplantation assays.

C-kit–enriched cells isolated from male donors were transduced with the lentiviral constructs containing *Tet2*, *Stag2*, or nontargeting sgRNA. We specifically used male donors for our experiments since *Stag2* is an X-linked gene and we were interested in modeling loss of Stag2 expression similar to what has been observed in patients with *STAG2* LOF mutations. Cells were spinfected with lentivirus at 37°C for 90 minutes at 2000 rpm, washed twice, and injected into animals within 6 hours after transduction.

The following sgRNAs targeting mouse genes were used in the study: Tet2 sgRNA: TCAGGGGCGATGATGTACAT; Stag2 sgRNA: TAACACACAAAGACAGTACG.

Please see Supplemental Methods for additional details of transplantation assays and mouse analysis.

### Generation of PDX models.

PDX#1 AML model was generated from bone marrow mononuclear cells of a patient with *STAG2*-mutant AML, confirmed by Dana-Farber Cancer Institute’s Rapid Heme Panel sequencing (STAG2 p.31012* VAF 0.92; ASXL1 p.G642fs* VAF 0.513; NRAS p.G13D VAF 0.426; RUNX1 p.320* VAF 0.48). PDX#2 AML was generated from a skin lesion of a patient with *RAD21*-mutant AML (RAD21 p.R586* VAF 0.287; FLT3 p.D835Y VAF 0.514; RUNX1 p.Q265 VAF = 0.295; WT1 p.R301fs16* VAF 0.287; WT1 p.T377fs* VAF 0.278). See Supplemental Methods.

### In vivo drug treatment.

Two donor *Tet2*/*Stag2* and 2 donor *Tet2*/NTG mice were used to generate a cohort of mice for in vivo talazoparib experiments. Ten mice per arm were dosed with 0.25 mg/kg talazoparib or vehicle in 0.5% methylcellulose once daily by oral gavage. For drug treatment of PDX models, the mice were dosed with 0.25 mg/kg talazoparib or vehicle in 0.5% methylcellulose (*n* = 9–10 mice/arm for *STAG2*-mutant PDX, *n* = 4 mice/arm for *RAD21*-mutant PDX). See Supplemental Methods.

### Indel sequencing analysis.

All sequencing analyses were performed on DNA extracted from 1 million murine bone marrow cells using the QIAamp DNA Micro Kit (Qiagen 56304). A sequential PCR was used to amplify the predicted Cas9 cut sites at both the *Tet2* and *Stag2* loci, as previously described, and all primers are listed below (47). NGS using the MiSeq desktop sequencer (Illumina) was performed, and 300 bp single-end reads were used to identify indels. The depth of sequencing was above 10,000 reads for each gene. CRISPR-Seq, a publicly available method on Terra (https://terra.bio/), a cloud-based genomic analysis platform, was used to detect indels. The pipeline has been previously described (47). Reads were mapped against mouse genome build Mm10.

The following sequencing primers were used. For mTet2 sgRNA (TCAGGGGCGATGATGTACAT): *FORWARD 5′- Sequencing Primer - Genomic Primer - 3′* ACACTCTTTCCCTACACGACGCTCTTCCGATCT GGTCACCCTCAATAGAGAAGACA *REVERSE 5′- Sequencing Primer – Genomic Primer - 3′* GTGACTGGAGTTCAGACGTGTGCTCTTCCGATCT TGGGCAGCTCTCCTATCCTT. For mStag2 sgRNA(TAACACACAAAGACAGTACG): *FORWARD 5′- Sequencing Primer - Genomic Primer - 3′* ACACTCTTTCCCTACACGACGCTCTTCCGATCTTTGGCTGCATAATAATAGCCTAAAC *REVERSE 5′- Sequencing Primer – Genomic Primer - 3′* GTGACTGGAGTTCAGACGTGTGCTCTTCCGATCT AGTTGATGACTGCTTTGGTAAATG.

### Hi-C methods and data processing.

Hi-C was performed as described previously (40) with some minor modifications. Please see Supplemental Methods for details. The data have been deposited in NCBI’s Gene Expression Omnibus (and are accessible through GEO Series accession number GSE165038).

### Statistics.

A *P* value less than 0.05 or an adjusted *P* value/FDR of less than 0.05 was considered significant. Statistical significance of differences in cohesion defects between WT and single and double cohesin knockout cells was determined using a 1-way ANOVA (Figure 1B). Statistical significance of enrichment of DNA damage repair and replication proteins was determined using a 1-tailed Fisher’s exact test (Figure 1C). Statistical significance of differences in replication fork stalling between WT and single and double cohesin knockout cells was determined using a 2-tailed unpaired Student’s *t* test (Figure 1D). Statistical significance of differences in leukemia burden between mice treated with talazoparib versus vehicle was determined using a 2-tailed unpaired Student’s *t* test (Figure 2, D and E; and Figure 4, F and G). Statistical significance of differences in blood counts of Tet2/NTG and Tet2/Stag2 mice in the presence and absence of treatment with talazoparib was determined using 2-tailed unpaired Student’s *t* tests (Figure 3D and Figure 4C). Statistical significance of differences in *Stag2* and *Tet2* indel fraction in the bone marrow of Tet2/NTG and Tet2/Stag2 mice treated with talazoparib or vehicle was determined using 2-tailed unpaired Student’s *t* tests (Figure 4B). Statistical significance of differences in survival of *RAD21*-mutant PDX model treated with talazoparib or vehicle was determined using Kaplan-Meier survival analysis (Figure 4G). Statistical significance of differences in colocalizing coefficients of cohesin with PARP or RPA1 protein in *STAG2* WT and mutant cells was determined using 2-tailed unpaired Student’s *t* tests (Figure 5G).

### Study approval.

All mouse experiments were conducted according to an IACUC-approved protocol at the Broad Institute.

## Author contributions

ZT and BLE designed the research; ZT, EC, KL, MS, ADD, SAC, JD, and BLE supervised the research; ZT, ALV, RAG, MV, JMKB, AH, CCL, EM, CH, JEH, MD, KDP, SK, and JR performed the research; ZT, ALV, JMKB, AH, CCL, EM, CH, MD, KDP, JR, EC, SVV, MS, and EAM analyzed the data. ZT and BLE wrote the manuscript.

## Supplementary Material

Supplemental data

Supplemental Table 1

Supplemental Table 2

## Version 1 12/22/2020

In-Press Preview

## Version 2 02/08/2021

Electronic publication
