# Supplementary material for: Cohesin mutations alter DNA damage repair and chromatin structure and create therapeutic vulnerabilities in MDS/AML
Source: JCI Insight. 2021 Feb 8;6(3):e142149. doi: 10.1172/jci.insight.142149 (PMC7934867; doi:10.1172/jci.insight.142149)

SUPPLEMENTAL METHODS, FIGURES and TABLES

Cohesin mutations alter DNA damage repair and chromatin structure and create therapeutic vulnerabilities in MDS/AML

Zuzana Tothova[1,2*], Anne-Laure Valton[3], Rebecca A. Gorelov[2], Mounica Vallurupalli[1,2], John M. Krill-Burger[2], Amie Holmes[4], Catherine C. Landers[2], J. Erika Haydu[2], Edyta Malolepsza[2], Christina Hartigan[2], Melanie Donahue[2], Katerina D. Popova[2], Sebastian Koochaki[1], Sergey V. Venev[3], Jeanne Rivera[5], Edwin Chen[5], Kasper Lage[2], Monica Schenone[2], Alan D. D'Andrea[4], Steven A. Carr[2], Elizabeth A. Morgan[6], Job Dekker[3,7], Benjamin L. Ebert[1,2,7*]

SUPPLEMENTAL METHODS

Generation of cohesin-mutant single cell clones

Both U937 and K562 parental cell lines were confirmed to be wild type for all cohesin complex components and regulators based on RNA-Seq, and exome or genome sequencing data generated by the Cancer Cell Line Encyclopedia (CCLE) prior to development of any cohesin-mutant models(1). Of note, since *STAG2* is an X-linked gene which undergoes normal X-inactivation in females, patients with predicted loss-of-function (LOF) *STAG2* mutations are predicted to lack normal STAG2 expression. Therefore, in both cases of U937 cells and K562 cells, which both carry two copies of the X chromosome, we screened for presence of homozygous *STAG2* LOF mutations. U937- and K562-Cas9 expressing cells were first generated by lentiviral transduction of parental U937 and K562 cells with a lentiviral vector expressing the Cas9 nuclease under blasticidin selection (pLX-311Cas9; Addgene #96924). Each Cas9-expressing cell line was subjected to a Cas9 activity assay using a separate lentiviral transduction with a GFP reporter construct (pXPR-011; Addgene #59702) and only cell lines with >80% Cas9 activity as determined by flow cytometry were used for subsequent generation of single cell clones. To generate the single cell clones, we cloned sgRNAs targeting *STAG2*, *SMC3*, *RAD21* or non-targeting sgRNAs into a minimal backbone plasmid (Addgene #41824) and transfected into U937-Cas9 or K562-Cas9 cells using nucleofection (Lonza, Nucleofector II). We single cell sorted GFP+ cells into 96 well plates 36-48 hours after transfection, and grew up single cell clones, which we confirmed by DNA sequencing of the targeted locus, as well as Western blotting. Select cohesin-mutant clones were transduced with GFP or mCherry and luciferase containing lentiviral plasmids (lenti-GFP-Luc2, or lenti-mCherry-Luc2) and sorted for GFP or mCherry positivity for use in mouse transplant experiments and competition assays. Choice of sgRNAs was driven by observation of presumed pathogenic *STAG2* mutations in patients based on Rapid Heme Panel sequencing at the Dana-Farber Cancer Institute (DFCI cBioPortal) (2).

Western Blotting and Immunoprecipitation (IP)

Cells were washed in cold 1X PBS (Corning CellGro) and lysed for 15-30min on ice using NP-40 lysis buffer (150mM NaCl, 50mM Tris pH 7.5 (Thermo Fisher Scientific 15567027), 1% NP40 (Roche 11332473001), 5% glycerol (Sigma-Aldrich G5516), and 100X Halt Protease and Phosphatase Inhibitor Cocktail (Thermo Fisher Scientific 78447) in water). Lysates were quantified using Pierce BCA Protein Assay Kit (Thermo Fisher Scientific 23225) and reduced with 6X Laemmli SDS-Sample Buffer (Boston BioProducts BP-111R) and 10X NuPAGE Sample Reducing Agent (Thermo Fisher Scientific NP0009) for 5min at 95°C.

For SMC1A IPs, protein lysates were incubated with SMC1A antibody overnight rotating at 4°C. 50µL of Protein G or A agarose beads in a 50% slurry (EZview Red Protein G Affinity Gel, Sigma-Aldrich, E3403 and EZview Red Protein A Affinity Gel, Sigma-Aldrich, P6486) were washed three times with NP-40 lysis buffer and incubated with the antibody and lysate mixture. The mixture was rotated for 2hrs at 4°C, and the beads were then washed once with NP-40 lysis buffer and twice with wash buffer (150mM NaCl, 50mM Tris pH 7.5 (Thermo Fisher Scientific 15567027), 1% glycerol (Sigma-Aldrich G5516), 100X Halt Protease and Phosphatase Inhibitor Cocktail (Thermo Fisher Scientific 78447) in water). Samples were reduced as stated above.

For Western blotting, samples were run on NuPAGE 4-12% Bis-Tris Protein Gels (Invitrogen NP0321 and NP0316) in NuPAGE MOPS SDS Running Buffer 20X (Life Technologies NP0001) or NuPAGE MES SDS Running Buffer 20X (Life Technologies NP0002) and transferred onto Nitrocellulose Pre-Cut Blotting Membranes (0.45µm, Invitrogen LC2001) using 10X Tris/Glycine

Buffer (Bio-Rad 1610734) and 20% Methanol (VWR TXBJ349664LBRI) in water. Gels were transferred at 90V for 2-2.5hrs or 40V overnight at 4°C. Membranes were blocked with 5% non-fat dry milk (Bio-Rad 1706404XTU) in Tris Buffered Saline with Tween-20 (Cell Signaling Technology, 9997) for 30-60min at room temperature. Incubation with primary antibody was performed overnight at 4°C or for 2-4hrs at room temperature in 5% milk in TBST. Primary antibodies used for characterization of control and *STAG2* knockout clones are as follows: Phospho-Histone H2A.X, STAG2, anti-SA1, and anti-SMC1. Membranes were washed 3 times for 5min each in TBST and incubated in Anti-rabbit IgG, Anti-mouse IgG, or Rabbit anti-Goat IgG secondary as needed in 5% milk in TBST for 1hr at room temperature. Membranes were washed an additional 3 times for 5min each in TBST and visualized after a 5min incubation with SuperSignal West Dura Extended Duration Substrate (Thermo Scientific, 34075), SuperSignal West Pico Chemiluminescent Substrate (Thermo Fisher Scientific, 34078), or Amersham ECL Prime Western Blotting Detection Reagent (GE Healthcare, RPN2232). Additional blots were imaged using the LI-COR platform (LI-COR Biosciences) with IRDye 800CW and IRDye680RD secondary antibodies (mouse, IRDye 680LT Donkey-anti-mouse IgG(925-68022) used at 1:15,000; rabbit, IRDye 800CW Goat anti-rabbit IgG (926-32211) used at 1:10,000) and imaging was performed on an Odyssey CLx Infrared Imaging System.

To assess levels of DNA damage, cells were treated with 100nM Talazoparib (Selleck S7048) and incubated at 37°C for 24hrs before harvesting protein lysate. Cells were also irradiated using an X-ray irradiator at 100rads or 10Gy, and protein lysate was harvested after 1hr at 37°C. Samples were run on NuPAGE 3-8% Tris-Acetate Protein Gels (Thermo Fisher Scientific, EA0375) and transferred as above at 90V for 2hrs at 4°C. Membranes were blocked for 30min at room temperature and incubated with the primary antibody overnight at 4°C. Primary antibodies used are as follows: ATR, Chk1, Phospho-Chk2, and Vinculin in 5% milk in TBST; Anti-Chk2 in 1% milk in TBST; Phospho-ATR, ATM , Phospho-ATM, and Phospho-Chk1 in 5% BSA in TBST. Membranes were washed 10 times for 10min each and incubated with the secondary antibody as stated above for 1hr at room temperature. After incubation, membranes were washed an additional 10 times for 10min each and visualized as above.

The following antibodies were used in the study:
Anti-ATR (Abcam, Cat#ab54793)
Anti-SA1 (Abcam, Cat#ab4455)
Anti-SMC1 (Abcam, Cat#ab140493)
STAG2 (Cell Signaling Technology, Cat#4239)
β-Actin, 8H10D10 (Cell Signaling Technology, Cat#3700)
Phospho-Histone H2A.X, Ser139, 20E3 (Cell Signaling Technology, Cat#9718)
Chk1, 2G1D5 (Cell Signaling Technology, Cat#2360)
Phospho-Chk2, Th468, C13C1 (Cell Signaling Technology, Cat#2197)
Phospho-ATR, Ser428, D6H9 (Cell Signaling Technology, Cat#2853)
ATM, 11G12 (Cell Signaling Technology, Cat#92356)
Phospho-ATM, Ser1981, D6H9 (Cell Signaling Technology, Cat#5883)
Phospho-Chk1, ser345, 133D3 (Cell Signaling Technology, Cat#2348)
Vinculin, E1E9V XP (Cell Signaling Technology, Cat#13901)
Anti-rabbit IgG, HRP-linked (Cell Signaling Technology, Cat#7074)
Anti-mouse IgG, HRP-linked (Cell Signaling Technology, Cat#7076)
Anti-Chk2, clone 7 (EMD Millipore, Cat#05-649)
Rabbit anti-Goat IgG, HRP conjugate (EMD Millipore, Cat#AP106P)

Genome-wide CRISPR screening

5 *STAG2*-mutant and 6 *STAG2*-wild type U937 cells expressing Cas9 and confirmed to have >80% Cas9 activity by the GFP reporter construct (pXPR-011; Addgene #59702) were infected with the genome-wide human Avana LentiGuide-Puro CRISPR library (Broad Genetic Perturbations Platform), which contains ~75,000 sgRNAs targeting ~19,000 genes and 1000 controls, in two separate experiments, as previously described (3). Briefly, cells were selected in blasticidin for 14 days to maximize Cas9 expression. Blasticidin was washed out of the media two days prior to transduction. Puromycin selection of transduced cells was initiated on Day 2 after transduction and carried out for a total of 5 days. Blasticidin was restarted on Day 8 after transduction and included in the media through the end of the experiment. Cell pellets were collected on Day 21, genomic DNA was purified and the sgRNA sequences amplified with sufficient gDNA to maintain representation, and quantified using next generation sequencing.

Transplantation assays and mouse analysis.

8-10 week old male and female NSGS mice (NOD-SCID; IL2Rγ null; Tg(IL3, CSF2, KITL)) obtained from The Jackson laboratory, strain 013062) were sublethally irradiated using Gamma-cell irradiator (Best Theratronics) at a dose of 250 Rads, and retro-orbitally injected with 200,000-1 million cells/mouse. 4-10 recipients were injected per arm. Animals were monitored daily for presence of disease, and were sacrificed at designated times after transplantation or when moribund. Peripheral blood was collected from the retro-orbital cavity using a heparinized glass capillary and automated total and differential blood cell counts were determined using ADVIA Hematology system (Bayer). Collected blood was also used to prepare blood smears, which were stained with May Gruenwald and Modified Giemsa (Sigma Aldrich). Following sacrifice, mice were examined for presence of tumors, enlarged lymph nodes or other abnormalities, and organs were collected for further cell and histopathologic analysis. Single cell suspensions were made from bone marrow, and spleen, washed, red blood cells were lysed, and samples were frozen in 10% dimethylsulfoxide/ 90% FCS until analysis. Peripheral blood and bone marrow from mice at the time of sacrifice were analyzed for contribution of human CD45+ hematopoiesis by flow cytometry (CD45-PE-Cy7 antibody (BD Biosciences, clone HI30)) as well as immunohistochemistry (IHC). All IHC was performed on the Leica Bond automated staining platform. Antibody against human CD33 from Leica, catalogue # NCL_CD33, clone PWS44, was run at 1:100 dilution using the Leica Biosystems Refine Detection Kit with EDTA antigen retrieval. Antibody against human CD34 from Beckman Coulter, catalogue # IM0787, clone QBEnd10, was run at 1:70 dilution using the Leica Biosystems Refine Detection Kit with citrate antigen retrieval. Antibody against CD45/LCA from Dako, catalogue # M0701, clone 2B11 + PD7/26, was run at 1:50 dilution using the Leica Biosystems Refine Detection Kit with citrate antigen retrieval. Antibody against F4/80 from CST, catalogue # 70076, clone D2S9R, was run at 1:500 dilution using the Leica Biosystems Refine Detection Kit with citrate antigen retrieval. For sequential syngeneic transplant experiments, 100,000 c-kit enriched cells or 1-2 million bone marrow cells were transplanted into sublethally irradiated SJL mice. 6-20 recipient mice were transplanted per genotype across multiple independent experiments.

Generation of patient-derived xenograft (PDX) models. All PDX models were generated using 11-18 week old female NSGS mice (NOD-SCID; IL2Rγ null; Tg(IL3, CSF2, KITL), The Jackson laboratory, strain 013062), which were irradiated using an X-ray irradiator with 200 rads 24 hours prior to transplant.

The *STAG2*-mutant AML PDX model (PDX#1 AML) was originally generated from the unsorted mononuclear cells of a bone marrow aspirate from a patient with *STAG2*-mutant AML, confirmed

by Dana-Farber Cancer Institute's Rapid Heme Panel sequencing analysis (STAG2 p.31012* VAF 0.92; ASXL1 p.G642fs* VAF 0.513; NRAS p.G13D VAF 0.426; RUNX1 p.320* VAF 0.48) . These cells were thawed, resuspended in Hanks' Balanced Salt Solution (Thermo Fisher Scientific 14025076), and injected retro-orbitally into two NSGS mice ("P0"). The mice were monitored daily, and sacrificed after two months once they became moribund. Cells were harvested according to the same protocol described above, and human myeloid disease was confirmed using NGS, flow cytometry, and immunohistochemistry. The presence of human cells was confirmed by flow cytometry on bone marrow using the following antibodies: anti-CD33 (BioLegend, 366611), anti-CD45 (BD Biosciences, 557748), anti-CD34 (BD Biosciences, 562577). In addition to haematoxylin and eosin (H&E) staining, immunohistochemistry analyses were similarly performed using the following antibodies: anti-CD45 (), anti-CD33 (), anti-CD34 (). May Gruenwald and Modified Giemsa (Sigma Aldrich) was used to stain bone marrow cytospins (20,000 cells/cytospin). The viably frozen bone marrow cells were serially injected (1 million cells per mouse by tail vein injection) to generate "P1" progeny (n=5). These mice were similarly monitored daily, sacrificed once they became moribund after 5 months, and human disease was confirmed as described above. Cells from the bone marrow, spleen and peripheral blood were viably frozen to use for further analyses and transplantation as described below.

The *RAD21*-mutant AML PDX model (PDX#2 AML) was generated from a skin lesion of a patient with *RAD21*-mutant AML (RAD21 p.R586* VAF 0.287; FLT3 p.D835Y VAF 0.514; RUNX1 p.Q265 VAF=0.295; WT1 p.R301fs16* VAF 0.287; WT1 p.T377fs* VAF 0.278). The initial sample that had been serially transplanted twice in mice, once under the renal capsule, and subsequently by subcutaneous injection, was acquired from PRoXe (https://www.proxe.org/), and mutation status was confirmed using the Dana-Farber Cancer Institute's Rapid Heme Panel (RAD21 p.R586* VAF 0.484; FLT3 p.D835Y VAF 0.989; RUNX1 p.Q265 VAF=0.51; WT1 p.V368fs16* VAF 0.434; WT1 p.T377fs* VAF 0.544). The sample was propagated intravenously for *in vivo* drug studies and *RAD21* mutation status was confirmed by Sanger sequencing.

In vivo drug treatment of AML cell line xenograft models

For *in vivo* talazoparib experiments performed with AML cell lines, NSGS recipient mice were sublethally irradiated using the X-ray irradiator (Rad Source) at a dose of 200 Rads and injected by tail vein with 1 million GFP+ or mCherry+ *STAG2*-mutant or WT U937 cells, individually (5 mice per arm) or as a 1:1 mixture (10 mice per arm), and dosed with 0.25mg/kg talazoparib or an equivalent amount of vehicle in 0.5% methylcellulose solution daily by oral gavage starting on Day 7 for 14-19 days until disease development, at which point they were sacrificed. Mice were weighed daily, and the daily dose was not administered if the body weight dropped by 15% from the day before. Animals were monitored daily for presence of disease, and were sacrificed when moribund. Following sacrifice, mice were examined for presence of tumors, enlarged lymph nodes or other abnormalities, and organs were collected for further cell and histopathologic analysis. Single cell suspensions were made from bone marrow, and spleen, washed, red blood cells were lysed, and samples were frozen in 10% dimethylsulfoxide/ 90% FCS until analysis.

In vivo drug treatment of PDX and Syngeneic Mouse Models.

Cells pooled from two donor *Tet2*/*Stag2* or two donor*Tet2*/NTG mice were used to generate a cohort of mice for *in vivo* talazoparib experiments. The mice were sacrificed and harvested as described above. Whole bone marrow cells were injected into 8 week old female SJL mice (B6.SJL-Ptprc) obtained from The Jackson laboratory, strain 002014) that had been lethally irradiated 14 and 7 hours prior to transplantation. They were injected retro-orbitally with 2 million cells/mouse from the *Tet2/Stag2* sample (n=20) or the *Tet2*/NTG sample (n=20). Engraftment was monitored in the peripheral blood by flow cytometry tracking both fluorescent reporters as

well as CD45.1 and CD45.2 positivity using the following antibodies: anti-CD45.1 (BioLegend, 110713) and anti-CD45.2 (BioLegend109805). After two months, once the percentage of CD45.2+ cell reached 50% in the peripheral blood, half of the mice within each group (n=10 for both *Tet2/Stag2* and *Tet2*/NTG) were dosed with 0.25mg/kg talazoparib in 0.5% methylcellulose once daily by oral gavage, while the other half (n=10 for both *Tet2/Stag2* and *Tet2*/NTG) were dosed with an equivalent amount of vehicle in 0.5% methylcellulose solution.

Both PDX#1 AML and PDX#2 AML models described above were used for the *in vivo* talazoparib experiments. 24 hours prior to the transplant, 11-15 week old NSGS mice (NOD-SCID; IL2Rγ null; Tg(IL3, CSF2, KITL) obtained from The Jackson laboratory, strain 013062) were irradiated with 200 rads using an X-ray irradiator. To generate the *STAG2*-mutant AML PDX model (PDX#1 AML model) for *in vivo* drug treatment, 19 sublethally NSGS mice were injected by tail vein with 2,275,000 viably frozen spleen cells/mouse from a previously described "P1" mouse. To generate the *RAD21*-mutant AML PDX model, 8 sublethally irradiated NSGS mice were injected by tail vein injection with 1,300,000 viably frozen cells. The presence of human disease in the peripheral blood of both PDX models was monitored by flow cytometry using the following antibodies: anti-CD33 (BioLegend, 366611), anti-CD45 (BD Biosciences, 557748), anti-CD34 (BD Biosciences, 562577). Once the hCD45 positivity was above 1% in the peripheral blood, 2 and 3 months after transplant for the *RAD21*-mutant and *STAG2*-mutant AML models respectively, the mice were divided into two arms: one arm (n=10 for the *STAG2*-mutant AML, n=4 for the *RAD21*-mutant AML) was dosed with 0.25mg/kg talazoparib in 0.5% methylcellulose, while the other arm (n=9 for the *STAG2*-mutant AML, n=4 for the *RAD21*-mutant AML) was dosed with an equivalent amount of vehicle in 0.5% methylcellulose solution.

For all *in vivo* drug treatments, the mice were dosed daily by oral gavage. Mice were weighed daily, and the dose was not administered if the body weight dropped by 15% from the starting mouse weight. Mice were monitored on a daily basis, and were sacrificed when moribund or at a designated time after transplant. Relevant tissues were harvested as above.

Flow cytometry

U937 and K562 cells were single cell sorted with a FACSAriaII instrument (Becton Dickinson, Mountain View, CA) after DAPI staining for viability (Thermo Fisher Scientific). mCherry+ and GFP+ cohesin-mutant cells used for *in vivo* transplant studies and *in vitro* competition assays were sorted with a MoFlo Astrios EQ sorter (Beckman Coulter) or Sony SH800S Sony Cell Sorter. Readout of *in vitro* competition assays was perfomed using CytoFLEX (Beckman Coulter).

***In vitro* drug treatment and *in vitro* competition assays**

Talazoparib was obtained from Selleck (S7048) and dissolved in DMSO. All drug dose response assays were conducted in 96 well plates. For cohesin-mutant cell lines, 5000 cells were plated per well. Cells were split 1:4 every 4 days and redosed with fresh medium supplemented with fresh drug every 4 days.

Drug dose response assays on patient samples were conducted on viably frozen bone marrow aspirates from patients with *STAG2*-wild type or mutant AML, acquired from the Pasquarello Tissue Bank at Dana Farber Cancer Institute. The following samples were used -- patient 1: 64-year old male with AML, BCOR S1439fs*(VAF 0.12), DNMT3A P420fs*(VAF 0.39), DNMT3A V895M (VAF 0.39), IDH2 R172K (VAF 0.42), RUNX1 V124fs* (VAF 0.39); patient 2: 50-year old male with AML, IDH2 R140Q (VAF 0.45), DNMT3A R882H (VAF 0.55); patient 3: 76-year old male with AML, ASXL1 G642fs* (VAF 0.24), IDH1 p.R132L (VAF 0.04), RUNX1 Q390fs* (VAF 0.09), STAG2 T514fs* (VAF 0.19); patient 4: 65-year old male with AML, IDH2 R140Q VAF(0.39),

RUNX1 A251Sfs* (VAF 0.76), STAG2 c.2096+2_2096+3insCTTCCT(splice)(VAF 0.59). After thawing, cells were cultured for 24 hours in StemSpan™ Serum-Free Medium (Stem Cell Technologies, 09650) supplemented with rh-SCF (100ng/mL, (Miltenyi Biotec 130-096-695)), rh-FLT3-L (100ng/mL, (Miltenyi Biotec 130-093-909)), rh-IL3 (25ng/mL, (Miltenyi Biotec 130-093-909)), rh-IL6 (25ng/mL, (Miltenyi Biotec, 130-095-352)), rh-G-CSF (25ng/mL, (Miltenyi Biotec 130-093-861)), rh-TPO (15ng/mL, (Miltenyi Biotec, 130-094-013)) and Penicillin-Streptomycin-Glutamine (Thermo Fisher Scientific 10378016). 20,000 cells were plated per well, and viable cell number was measured every 3 days using the CellTiter-Glo luminescent cell viability assay (Promega). 3 technical replicates were performed across various drug concentrations of talazoparib for each patient sample. For all drug dose response assays, viability was normalized to DMSO-treated wells.

For competition experiments, GFP- and mCherry- labeled cohesin-mutant cells were mixed in 1:10 and 1:100 ratios, and plated in 96 well plates. Cells were passaged and split 1:4 every four days and redosed with fresh medium supplemented with fresh drug every 4 days. Fraction of the cells was stained for viability with DAPI, and % mCherry+DAPI- and GFP+DAPI- cells were determined using flow cytometry. Drug dosing for all experiments performed in 96 well plates was performed using the D300e drug dispenser (Tecan). 3 technical replicates were performed.

DNA fiber assay

Briefly, 1 × 10^5 U937-*STAG2*-wild type or mutant cells were seeded in a well of a 96-well plate in a volume of 100 µl. Replicating DNA was first labeled with 25 µM IdU (Sigma), followed by 250 µM CldU) (Sigma) for 20 min each. Cells were then harvested, genomic DNA was extracted in fiber lysis buffer (200 mM Tris-HCl pH 7.5, 50 mM EDTA, 0.5% SDS), and individual DNA molecules were stretched on glass slides using a 15° tilting method. The slides were immunostained with anti-BrdU (BD Bioscience) (1:25) and anti-BrdU (Abcam) (1:400) in blocking solution, washed and stained with sheep anti-mouse Cy3 (Sigma) (1:500) and goat anti-rat AF488 (Invitrogen) (1:400). Slides are washed three times with PBS and analyzed under oil immersion on a Zeiss Axioscope 2 fluorescence microscope.

Replication fork stalling, asymmetry and fork rate

The length of the fibers were measured in micrometers and converted to kilobases according to a constant stretching factor of 1 µM = 2 kb, as previously reported(4). A stalled fork is defined as a >30% reduction in fork progression in the second labelling step relative to the first. An asymmetrical origin is defined as a ratio between the two oppositely moving arms (arbitrarily designed “left” and “right”) of the origin structure as <0.7. Testing for statistical significance was performed on the ratio between the two arms using a single-sample t test using a predicted population mean of 1. Fork rate was calculated by measuring the combined red and green tracts or progressing structures and dividing by the total labeling time (40 min).

Cohesion defect analysis

STAG2-mutant and WT cells were transduced with lentiCRISPRv2 plasmid containing STAG1-sgRNA or control sgRNAs, puromycin selected and cohesion defect analysis was performed 4 days after transduction. Cells were exposed for 2 hr to 100 ng/ml colcemid, treated with a hypotonic solution (0.075 M KCl) for 20 min, and fixed with 3:1 methanol/acetic acid. Slides were stained with Giemsa stain and 100 metaphase spreads were scored for aberrations in a blinded fashion. The number of metaphase spreads with railroad chromosomes, premature centromere separation or combination of both was calculated for each condition.

Super-resolution microscopy

STAG2-mutant and wild type U937 cells were cytospun on pre-cleared glass cover slips (CytoSpin4, Thermo Fisher) and fixed in 4% paraformaldehyde (VWR, BT140770) in PBS for 10min at RT. They were washed with PBS for 5 min three times, followed by permeabilization with 0.5% Triton X100 (Sigma Aldrich, X100) in PBS for 10 minutes at RT. Following three washes in PBS for 5 min, cells were blocked with 4% goat serum in PBS (Vector Laboratories, S-1000) for 1 hr at RT and incubated with primary antibodies (anti-SMC1A Abnova MAB10393 1:200 dilution; anti-PARP1 Cell Signaling 9532 1:800 dilution; anti-RPA1 CST 2267 1:100 dilution) in 4% goat serum overnight at 4C. After three washes in PBS, coverslips were incubated with secondary antibodies (Goat anti-mouse IgG Alexa Fluor 488 Thermo Fisher A11029 1:500 dilution, and Goat anti-Rabbit IgG Alexa Fluor 594 Invitrogen A11037 1:500 dilution) in the dark for 1 hour at RT. Cells were washed three times with PBS and 20µm/ml Hoechst 33258 (Life Technologies, H3569) was used to stain nuclei for 5 min at RT in the dark. Glass slides were mounted onto slides with Prolong Diamond Antifade 5 (Thermo Fisher, P36961) for 24 hours at RT. Coverslips were sealed with transparent nail polish (Electron Microscopy Science Nm, 72180) and stored at 4°C until image acquisition. Images were acquired on the ELYRA super-resolution microscope with a 100x objective using Zeiss ZEN Black software, with 5-10 Z-stacks 80-100nm apart. Images were post-processed using the Zeiss ZEN Blue software.

Super-resolution microscopy – colocalization analysis

Colocalization analysis was performed using the Zeiss ZEN blue software (https://www.zeiss.com/content/dam/Microscopy/Downloads/Pdf/FAQs/zen-aim_colocalization.pdf). 15-20 cells were analyzed in 5-10 Z-stacks per sample and 2-4 biological replicates were used in each experiment. Colocalization coefficients were determined based on (5) and calculated for each channel as a ratio of the sum of pixels in the colocalized quadrant and the sum of pixels in the non-colocalized and colocalized quadrants.

Hi-C methods

Hi-C was performed as described previously(6) with some minor modifications.

Cell lysis

10 million formaldehyde cross-linked cells were incubated in 1000 µl of cold lysis buffer (10 mM Tris-HCl pH8.0, 10 mM NaCl, 0.2% (v/v) Igepal CA630, mixed with 10 µl of 10X protease inhibitors (Thermofisher 78438)) on ice for 15 minutes. Next, cells were lysed with a Dounce homogenizer and pestle A (Kimble Kontes # 885303-0002) by moving the pestle slowly up and down 30 times, incubating on ice for one minute followed by 30 more strokes with the pestle. The suspensions were centrifuged for 5 minutes at 2,000 g at RT using a table top centrifuge (Centrifuge 5810R, (Eppendorf). The supernatants were discarded and the pellet were washed twice with ice cold 500 µl 1x NEBuffer 3.1 (NEB). After the second wash, the pellets were resuspended in 720 uL of 1x NEBuffer 3.1 and split into two tubes. 18 uL were kept at -20°C to assess the chromatin integrity later. Chromatin was solubilized by addition of 38 µl 1% SDS per tube and the mixture was resuspended and incubated at 65°C for 10 minutes. Tubes were put on ice and 43 µl 10% Triton X-100 was added.

Chromatin digestion

Chromatin was digested by adding 400 Units DpnII (NEB) per tube at 37°C for overnight digestion with alternating rocking. Digested chromatin samples were incubated at 65°C for 20 minutes to

inactivate the DpnII enzymes, spun shortly and transferred to ice. 10 uL were kept at -20°C to assess the digestion efficiency later.

Biotin fill-in
DNA ends were marked with biotin-14-dATP by adding 60 µL of biotin fill-in master mix (1XNEB 3.1, 0.25 mM dCTP, 0.25 mM dGTP, 0.25 mM dTTP, 0.25 mM biotin-dATP (ThermoFisher#19524016), 50U Klenow polymerase Polymerase I (NEB M0210L)). The samples were incubated at 23°C for 4 hours with some agitation and then placed on ice.

Blunt end ligation
Ligations were performed by adding 665 µL of ligation mix (240 µL of 5x ligation buffer (1.8X) (Invitrogen), 120 µL 10% Triton X-100, 12 µL of 10 mg/mL BSA, 50 µL T4 DNA ligase (Invitrogen 15224090), and 243 µL ultrapure distilled water (Invitrogen)). The reactions were then incubated at 16°C for 4 hours with some agitation. After the ligation, the crosslink was reversed by adding 50 uL of 10mg/mL proteinase K (Fisher BP1750I-400) and incubated at 65°C for 2 hours followed by a second addition of 50 µL 10 mg/mL Proteinase K and overnight incubation at 65°C.

DNA purification
Reactions were cooled to room temperature and the DNA was extracted by adding an equal volume of saturated phenol pH 8.0: chloroform (1:1) (Fisher BP1750I-400), vortexing for 1 minute, transferred to a phase-lock tube and spun at 16,000g for 5 minutes. DNA was precipitated by adding 1/10th of 3 M sodium acetate pH 5.2, 2 volumes of ice-cold 100% ethanol and incubated for at least an hour at -80°C. Next, the DNA was pelleted at 16,000 g at 4°C for 30 minutes. The supernatants were discarded, the pellets were dissolved in 500 µL 1X TLE and transferred to a 15 mL AMICON Ultra Centrifuge filter (UFC903024 EMD Millipore). 10 mL of TLE was added to wash the sample, the columns were spun at 4,000 g for 10 minutes and the flow-throughs were discarded. A second wash with 10 mL of TLE was done and the sample was transferred to a 0.5 mL AMICON Ultra Centrifuge filter (UFC5030BK EMD Millipore) and spin at 16,000g for 10 minutes to reduce the sample to 50 uL. RNA was degraded by adding 1 µL of 10 mg/mL RNAase A and incubated at 37°C for 30 minutes. DNA was quantified by loading on a 1% gel 1 uL of the Hi-C sample, the chromatin integrity and the digestion controls.

Biotin removal from unligated ends
To remove biotinylated nucleotides at DNA ends that did not ligate, the Hi-C samples were treated with T4 DNA polymerase. 5 µg of Hi-C library were incubated with 5 µL 10x NEBuffer 3.1, 0.025 mM dATP, 0.025 mM dGTP and 15 U T4 DNA polymerase (NEB # M0203L) in 50 uL. Reactions were incubated at 20°C for 4 hours, the enzymes were then inactivated at 75°C for 20 minutes and placed at 4°C.

DNA shearing
The samples were pooled and the volume was brought up to 130 µL 1X TLE. The DNA was sheared to a size of 100-300 bp using a Covaris instrument (Duty Factor 20%, Cycles per Burst 200, peak power 50, average power 17.5 and process time 180 sec). The volume was brought up to 500 µL with TLE for Ampure fractionation. To enrich for DNA fragments of 100-300 bp an Ampure XP fractionation was performed (Beckman Coulter, A63881) and the DNA was eluted with 50 µL of water. The size range of the DNA fragments after fractionation was checked by running an aliquot on a 2% agarose gel.

End repair
To proceed for end repair, 45 µL of Hi-C sample was transferred to a PCR tube, then 25 µL of the end-repair mix (3.5X NEB ligation buffer (NEB B0202S), 17.5 mM dNTP mix, 7.5 U T4 DNA

polymerase (NEBM0203L), 25 U T4 polynucleotide kinase (NEB M0201S), 2.5 U Klenow polymerase Polymerase I (NEB M0210L)) was added. The reactions were then incubated at 37°C for 30 minutes, followed by incubation at 75°C for 20 minutes to inactivate Klenow polymerase.

Biotin pull down

To pull down biotinylated DNA fragments, 50 µL of MyOne streptavidin C1 beads mix (Thermo Fisher 65001) was transferred to a 1.5 mL tube. The beads were washed twice by adding 400 µL of TWB (5 mM Tris-HCl pH8.0, 0.5 mM EDTA, 1 M NaCl, 0.05% Tween20) followed by incubation for 3 minutes at RT. After the washes, the beads were resuspended in 400 µL of 2X Binding Buffer (BB) (10 mM Tris-HCl pH8, 1 mM EDTA, 2 M NaCl) and mixed with the 400 µL DNA from the previous step in a new 1.5 mL tube. The mixtures were incubated for 15 minutes at RT with rotation. The DNA bound to the beads was washed first with 400 µL of 1X BB and then with 100 µL of NEB2.1 1X. Finally, the DNA bound to the beads was eluted in 41 µL of NEB2.1 1X.

A-tailing

Then, dATP was added to the 3' ends by adding 9 µL of A-tailing mix (5 µL NEB buffer 2.1, 5 µL of 1 mM dATP, 3 U Klenow exo (NEB M0212S)) to the 41 µL of DNA sample from the previous step. The reaction was incubated in a PCR machine (at 37°C for 30 minutes, then at 65°C for 20 minutes, followed by cool down to 4°C). Next, the tube was placed on ice immediately. The streptavidin beads bound to DNA were washed twice using 100 µL 1X T4 DNA Ligase Buffer (Invitrogen). Finally, streptavidin beads bound to DNA were resuspended in 36.25 µL 1X T4 DNA Ligase buffer (Invitrogen).

Illumina adapter ligation and PCR

The TruSeq DNA LT kit Set A (REF#15041757) was used. 10 µL of ligation mix (3 µL Illumina paired-end adapters, 4 µL T4 DNA ligase Invitrogen, 2.75 µL 5x T4 DNA ligase buffer (Invitrogen 5X)) was added to the 36.25 µL Hi-C sample from the previous step. The ligation samples were then incubated at room temperature for 2 hours on a rotator. The streptavidin beads bound to DNA were washed twice with 400 µl of TWB, then twice using 100 µL NEB2.1 1X. Finally, the samples were resuspended in 20 µL of NEB2.1 1X. Two trial PCR reactions (6 and 8 cycles) were performed as follows (0.9 µL DNA bound to beads, 1.5 µL of Primers mix (TruSeq DNA LT kit Set A 15041757), 6 µL Master Mix (TruSeq DNA LT kit Set A 15041757), 6.6 µL of ultrapure distilled water (Invitrogen)). The number of PCR cycles to generate the final Hi-C material for deep sequencing was chosen based on the minimum number of PCR cycles in the PCR titration that was needed to obtain sufficient amounts of DNA for sequencing. ClaI digestion was done as a library quality check. A downward shift of the amplified DNA to smaller sizes indicates that DNA ends were correctly filled in and ligated (creating a ClaI site). Primers were removed using Ampure XP beads. The libraries were sequenced using 50 bp paired end reads on a HiSeq4000. Four libraries were sequenced in one lane to assess the quality of the Hi-C library. For replicate 2, deeper sequencing was generated with 2 more lanes of sequencing.

Hi-C data processing

Hi-C libraries were processed using the distiller pipeline (https://github.com/mirnylab/distiller-nf). Briefly, reads were mapped to the human reference assembly hg19 using bwa mem to map fastq pairs in a single-side fashion (-SP). Alignments were parsed and pairs were classified using the pairtools package (https://github.com/mirnylab/pairtools) to generate pairs files. Uniquely mapped pairs were kept and duplicate pairs arising from PCR were removed. Pairs with high mapping quality scores on both sides (MAPQ > 30) were kept and aggregated into contact matrices in the cooler format using the cooler package(7). Data was binned and stored into multiresolution cooler files (1kb, 2kb, 5kb, 10kb, 25kb, 50kb, 100kb, 250kb, 500kb, 1Mb). All contact matrices were normalized using the iterative correction normalization(8) after ignoring the first 2 diagonals to

avoid short-range ligation artifacts at a given resolution. Low-coverage bins were excluded using the MADmax (maximum allowed median absolute deviation) filter on genomic coverage, described in (9), using the default parameters. The pooled WT libraries have a total of 1,114,862,985 mapped reads and the pooled SA2 KO libraries have a total of 1,079,935,647 mapped reads.

Scaling plots
Scaling plots represent the genome wide contact frequency as a function of genomic separation for all intra-chromosomal interactions. The scaling plots were normalized to unity at separation = 100 kb. The derivative of the scaling plot (slope) was calculated and smoothed using a Gaussian smoothing of 2 as described previously(10).

A/B compartments
A and B compartments were assigned using an eigenvector decomposition procedure(8) implemented in the cooltools package (https://github.com/mirnylab/cooltools). Eigenvector decomposition was performed on observed-over-expected cis contact matrices binned at 100 kb. The first eigenvector (E1) positively correlated with gene density were used to assign A and B compartment identity to each bin. Saddle plot and saddle strength were plotted and calculated using cooltools.
(https://github.com/hms-dbmi/hic-data-analysis-bootcamp/blob/master/notebooks/04_analysis_cooltools-eigenvector-saddle.ipynb). The distance corrected interaction bins were sorted based on their PC1 value in increasing order in a 50 by 50 bin matrix and the binning was done without quantile binning. Compartment strength was calculated as the ratio of (AA+BB)/(AB+BA) using the values from a 10 bin X 10 bin square starting from the corner (20% of the saddle plot data).

Insulation score
Insulation scores were calculated and TAD boundaries called using cooltools implementation of diamond insulation method(11)
(https://github.com/hms-dbmi/hic-data-analysis-bootcamp/blob/master/notebooks/05_analysis_cooltools-insulation-score.ipynb) with the diamond size of 250 kb using contact matrices binned at 25 kb. TAD boundaries with a strength > 0.3 were selected.

Mass Spectrometry

SMC1A_iTRAQ (Supplementary Fig. 2SC and 2SD)

20 million cells were used to generate 2mg input protein and immunoprecipitation was performed using 25ug SMC1A antibody or 25ug control IgG as described above. The beads from immunoprecipitation were washed once with IP lysis buffer and twice with IP wash buffer. The beads were resuspended in 20µL of wash buffer, followed by 90 µL digestion buffer (2 M urea, 50 mM Tris HCl) and then 2 µg sequencing grade trypsin was added, followed by 1 hour of shaking at 700 rpm. The supernatant was removed and placed in a fresh tube. The beads were then washed twice with 50 µl digestion buffer and combined with the supernatant. The combined supernatants were reduced (2 µl 500 mM dithiothreitol, 30 minutes, room temperature) and alkylated (4 µl 500 mM iodoacetamide, 45 minutes, dark), and a longer overnight digestion was performed: 2 µg (4 µl) trypsin, shaken overnight. The samples were then quenched with 20 µl 10% formic acid and desalted on 10 mg Oasis cartridges. Desalted

peptides were labeled with iTRAQ reagents according to the manufacturer's instructions (AB Sciex, Foster City, CA).

iTRAQ label 114: GC1-1 (Rep1), GC3-1 (Rep 2)
iTRAQ label 115: STAG2-A2(Rep1), STAG2-E10 (Rep 2)
iTRAQ label 116: SMC3-H10(Rep1), SMC3-H8 (Rep 2)
iTRAQ label 117: GC1-1(12) (Rep1), GC3-1(12) (Rep 2)

Peptides were dissolved in 30 µl 0.5 M TEAB pH 8.5 solution (Sigma-Aldrich) and labeling reagent was added in 70 µl of ethanol. After a 1-h incubation, the reaction was stopped with 50 mM Tris-HCl pH 7.5. Differentially labeled peptides were mixed and subsequently desalted on a 10 mg SepPak column. 50% of the sample was used for SCX fractionation as described in(13), with 6 pH steps (buffers- all contain 25% acetonitrile) as below:

1: ammonium acetate 50 mM pH 4.5,
2: ammonium acetate 50 mM pH 5.5,
3: ammonium acetate 50 mM pH 6.5,
4: ammonium bicarbonate 50 mM pH 8,
5: ammonium hydroxide 0.1% pH 9,
6: ammonium hydroxide 0.1% pH 11.

Empore SCX disk used to make StageTips as described(13).

Reconstituted peptides from each fraction were separated on an online nanoflow EASY-nLC 1000 UHPLC system (Thermo Fisher Scientific) and analyzed on a benchtop Orbitrap Q Exactive Plus mass spectrometer (Thermo Fisher Scientific). The peptide samples were injected onto a capillary column (Picofrit with 10 µm tip opening/75 µm diameter, New Objective, PF360-75-10-N-5) packed in-house with 20 cm C18 silica material (1.9 µm ReproSil-Pur C18-AQ medium, Dr. Maisch GmbH) and heated to 50 °C in column heater sleeves (Phoenix-ST) to reduce backpressure during UHPLC separation. Injected peptides were separated at a flow rate of 200 nl min^{-1} with a linear 120 min gradient from 100% solvent A (3% acetonitrile, 0.1% formic acid) to 30% solvent B (90% acetonitrile, 0.1% formic acid), followed by a linear 9 min gradient from 30% solvent B to 60% solvent B and a 1 min ramp to 90% B. The Q Exactive instrument was operated in the data-dependent mode acquiring higher-energy collisional dissociation (HCD) tandem mass spectrometry (MS/MS) scans (R = 17,500) after each MS1 scan (R = 70,000) on the 12 top most abundant ions using an MS1 ion target of 3×10^6 ions and an MS2 target of 5×10^4 ions. The maximum ion time utilized for the MS/MS scans was 120 ms; the HCD-normalized collision energy was set to 27; the dynamic exclusion time was set to 20 s; and the peptide match and isotope exclusion functions were enabled.

All mass spectra were processed using the Spectrum Mill software package v6.0 prerelease (Agilent Technologies), which includes modules developed by us for iTRAQ-based quantification. For peptide identification MS/MS spectra were searched against the human Uniprot database (UniProt.human.20141017.RNFISnr.150contams) to which a set of common laboratory contaminant proteins was appended. Search parameters included ESI-QEXACTIVE-HCD scoring parameters, trypsin enzyme specificity with a maximum of two missed cleavages, 40% minimum matched peak intensity, ± 20 ppm precursor mass tolerance, ± 20 ppm product mass tolerance, and carbamidomethylation of cysteines and iTRAQ labeling of lysines and peptide N termini as fixed modifications. Allowed variable modifications were oxidation of methionine, N-terminal acetylation, pyroglutamic acid (N-termQ), deamidated (N), pyro carbamidomethyl Cys (N-termC), with a precursor MH+ shift range of −18–64 Da.

Identities interpreted for individual spectra were automatically designated as valid by optimizing score and delta rank1-rank2 score thresholds separately for each precursor charge state in each liquid chromatography-MS/MS while allowing a maximum target-decoy-based false-discovery rate (FDR) of 1.0% at the spectrum level.

In calculating scores at the protein level and reporting the identified proteins, redundancy is addressed in the following manner: the protein score is the sum of the scores of distinct peptides. A distinct peptide is the single highest scoring instance of a peptide detected through an MS/MS spectrum. MS/MS spectra for a particular peptide may have been recorded multiple times, (i.e. as different precursor charge states, isolated from adjacent SCX fractions, modified by oxidation of Met) but are still counted as a single distinct peptide. When a peptide sequence >8 residues long is contained in multiple protein entries in the sequence database, the proteins are grouped together and the highest scoring one and its accession number are reported. In some cases when the protein sequences are grouped in this manner there are distinct peptides which uniquely represent a lower scoring member of the group (isoforms or family members). Each of these instances spawns a subgroup and multiple subgroups are reported and counted towards the total number of proteins. iTRAQ ratios were obtained from the protein-comparisons export table in Spectrum Mill. To obtain iTRAQ protein ratios the median was calculated over all distinct peptides assigned to a protein subgroup in each replicate.

SMC1A_TMT6 (Fig. 1C)

18 million cells were used to generate 4mg input protein and immunoprecipitation was performed using 31.5ug SMC1A antibody as described above. The beads from immunoprecipitation were washed once with IP lysis buffer, twice with IP wash buffer, then once with PBS. The beads were resuspended in 20µL of PBS, followed by 90 µL digestion buffer (2 M urea, 50 mM Tris HCl) and then 2 µg sequencing grade trypsin was added, followed by 1 hour of shaking at 700 rpm. The supernatant was removed and placed in a fresh tube. The beads were then washed twice with 50 µl digestion buffer and combined with the supernatant. The combined supernatants were reduced (2 µl 500 mM dithiothreitol, 30 minutes, room temperature) and alkylated (4 µl 500 mM iodoacetamide, 45 minutes, dark), and a longer overnight digestion was performed: 2 µg (4 µl) trypsin, shaken overnight. The samples were then quenched with 20 µl 10% formic acid and desalted on 10 mg Oasis cartridges.

Desalted peptides were labeled with TMT6 reagents Lot# RA230200 (Thermo Fisher Scientific) Peptides were dissolved in 25 µl fresh 100 mM HEPES buffer. The labeling reagent was resuspended in 42 µl acetonitrile and 10 µl added to each sample as described below. After 1 hour incubation the reaction was stopped with 8 µl 5 mM hydroxylamine.

TMT6 labeling was performed as follows: Rep 1 (WT-1, STAG2-KO3, STAG2-KO5), Rep 2(WT-2, STAG2-KO2, STAG2-KO6).

50% of the combined sample was used for basic reversed phase fractionation as described in (13) with 6 cuts as below:

1. 10% ACN
2. 15% ACN
3. 20% ACN
4. 35% ACN
5. 50% ACN
6. 80% ACN

The fractions were then concatenated int 3 combining fractions (1+4), (2+5) and (3+6) to create three fractions. Empore SDB disk used to make StageTips as described (13).

Reconstituted peptides from each fraction were separated on an online nanoflow EASY-nLC 1000 UHPLC system (Thermo Fisher Scientific) and analyzed on a benchtop Orbitrap Q Exactive Plus mass spectrometer (Thermo Fisher Scientific). The peptide samples were injected onto a capillary column (Picofrit with 10 µm tip opening/75 µm diameter, New Objective, PF360-75-10-N-5) packed in-house with 20 cm C18 silica material (1.9 µm ReproSil-Pur C18-AQ medium, Dr. Maisch GmbH) and heated to 50 °C in column heater sleeves (Phoenix-ST) to reduce backpressure during UHPLC separation. Injected peptides were separated at a flow rate of 200 nl min^{-1} with a linear 120 min gradient from 100% solvent A (3% acetonitrile, 0.1% formic acid) to 30% solvent B (90% acetonitrile, 0.1% formic acid), followed by a linear 9 min gradient from 30% solvent B to 60% solvent B and a 1 min ramp to 90% B. The Q Exactive instrument was operated in the data-dependent mode acquiring higher-energy collisional dissociation (HCD) tandem mass spectrometry (MS/MS) scans (R = 17,500) after each MS1 scan (R = 70,000) on the 12 top most abundant ions using an MS1 ion target of 3×10^6 ions and an MS2 target of 5×10^4 ions. The maximum ion time utilized for the MS/MS scans was 120 ms; the HCD-normalized collision energy was set to 29; the dynamic exclusion time was set to 20 s; and the peptide match and isotope exclusion functions were enabled.

All mass spectra were processed using the Spectrum Mill software package v6.0 prerelease (Agilent Technologies), which includes modules developed by us for TMT-based quantification. For peptide identification MS/MS spectra were searched against the human Uniprot database (UniProt.human.20141017.RNFISnr_CanCom.150contams) to which a set of common laboratory contaminant proteins was appended. Search parameters included ESI-QEXACTIVE-HCD scoring parameters, trypsin enzyme specificity with a maximum of two missed cleavages, 40% minimum matched peak intensity, ± 20 ppm precursor mass tolerance, ± 20 ppm product mass tolerance, and carbamidomethylation of cysteines and TMT6 labeling of lysines and peptide N termini as fixed modifications. Allowed variable modifications were oxidation of methionine, N-terminal acetylation, pyroglutamic acid (N-termQ), deamidated (N), pyro carbamidomethyl Cys (N-termC), with a precursor MH+ shift range of −18–64 Da. Identities interpreted for individual spectra were automatically designated as valid by optimizing score and delta rank1-rank2 score thresholds separately for each precursor charge state in each liquid chromatography-MS/MS while allowing a maximum target-decoy-based false-discovery rate (FDR) of 1.0% at the spectrum level.

Proteomics Analysis and graphing

Non-human proteins, proteins with less than two unique peptides, and proteins not present in the current HGNC database of protein coding genes (https://www.genenames.org/cgi-bin/statistics) were removed from further analyses. Ratios of intensities between channels were median normalized. Resulting data were analyzed and visualized using R (R Core Team, 2016). Statistical analyses were performed via moderated t-test from R package limma (14) to estimate p values for each protein and the false discovery rate corrections (FDR) were applied to account for multiple hypothesis testing. RNase A experiment data were statistically analysed using multi-sample t-test to account for different control samples treated with and without RNase. Figures were made using in-house written R scripts and library ggplot2. Pathways were taken from MSigDB database and statistical significance for enrichment was calculated using one-tailed Fisher exact test.

Negative selection CRISPR screen – Differential dependency analysis

Sequence of sgRNA barcodes was deconvoluted using PoolQ software to obtain raw barcode counts (http://portals.broadinstitute.org/gpp/public/dir/download?dirpath=protocols/screening&filename=4_Pooled_Screening_Deconvolution_using_PoolQ.pdf). All raw barcode count files are available at DOI (10.6084/m9.figshare.7120796; reviewer access at https://figshare.com/s/47ea327476fc114f853f).

Barcode reads are summed per sample across four sequencing lanes and annotated using the sgRNA to gene mapping in 'chip.txt' (available along with all other files referenced in this section at DOI (10.6084/m9.figshare.7120796; reviewer access at https://figshare.com/s/47ea327476fc114f853f)). The Avana library contains 1,351 sgRNAs with multiple perfect alignments to the genome, primarily due to paralogous gene families with high sequence identity and matches to non-protein coding regions, that are filtered out to reduce false positive dependencies. The number of perfect alignments between each Avana library sgRNA and the human genome were calculated using bowtie short read aligner as described (15) (alignments to chromosome X and Y are previously unpublished). Individual sgRNAs with greater than five perfect alignments to the genome or with suspected off-target activity (https://portals.broadinstitute.org/achilles/datasets/18/download/dropped_guides.txt) were dropped from further analyses. The complete Avana library targets 18,321 genes with 73,375 sgRNAs (~4 sgRNAs per gene). Post-filtering there are 18,106 genes targeted by 71,799 sgRNAs (at least 2 sgRNAs per gene). The summary counts table 'read_counts.txt' includes the 71,799 targeting sgRNAs along with 994 non-targeting sgRNA controls totally 72,793 total reagents.

Significance of differential gene dependencies between *STAG2*-mutant and *STAG2*-wild type cell lines was assessed using the MLE method from MAGeCK version 0.5.6 (16). The same pDNA was used across all samples, obviating the necessity for explicit batch correction, but per sample normalization was performed as part of the MLE metheod using the '--norm-method control --control-sgrna sgRNA_controls.txt' option where 'sgRNA_controls' are the non-targeting guides. The definition of the two-class comparison, *STAG2*-wild type samples (NCC4, NCB2A, NCB12, NCB1, NCE, NCF) as baseline class and *STAG2*-mutant samples (KOD5C, KOC5, KOG8B, KOA, KOG) as experimental class, was specified in the file 'design_mat.txt' and provided to the MLE method using the '-d design_mat.txt' option. The gene-level results are provided in the table 'two_class_MLE.gene_summary.txt' where the 'KO|wald-p-value' and 'KO|wald-fdr' columns are computed by the MAGeCK software with the permutation option set to '--permutation-round 100'. These results are listed in Supplementary Table S1.

Gene Set Enrichment

Genes were ranked by significance in the two-class differential dependency analysis and a one-sided Kolmogorov-Smirnov test was used to determine if a gene set is significantly enriched for differential dependencies. Gene sets include core complexes from CORUM protein complex release version 02.07.2017 (http://mips.helmholtz-muenchen.de/corum/#download) and MSigDB version 6 (http://software.broadinstitute.org/gsea/msigdb/collections.jsp) Hallmark, C1, C2:BioCarta, C2:KEGG, C2:Reactome, C3:TFT, and C5 sets.

SUPPLEMENTAL METHODS REFERENCES

1. Barretina J, Caponigro G, Stransky N, Venkatesan K, Margolin AA, Kim S, et al. The Cancer Cell Line Encyclopedia enables predictive modelling of anticancer drug sensitivity. *Nature.* 2012;483(7391):603-7.
2. Kluk MJ, Lindsley RC, Aster JC, Lindeman NI, Szeto D, Hall D, et al. Validation and Implementation of a Custom Next-Generation Sequencing Clinical Assay for Hematologic Malignancies. *J Mol Diagn.* 2016;18(4):507-15.
3. Doench JG, Fusi N, Sullender M, Hegde M, Vaimberg EW, Donovan KF, et al. Optimized sgRNA design to maximize activity and minimize off-target effects of CRISPR-Cas9. *Nat Biotechnol.* 2016;34(2):184-91.
4. Herrick J, and Bensimon A. Introduction to molecular combing: genomics, DNA replication, and cancer. *Methods Mol Biol.* 2009;521:71-101.
5. Manders EMMV, F. J.; Aten, J. A. Measurement of co-localization of objects in dual-colour confocal images. *Journal of Microscopy.* 1993;169:375-82.
6. Belaghzal H, Dekker J, and Gibcus JH. Hi-C 2.0: An optimized Hi-C procedure for high-resolution genome-wide mapping of chromosome conformation. *Methods.* 2017;123:56-65.
7. Abdennur N, and Mirny L. Cooler: scalable storage for Hi-C data and other genomically-labeled arrays. *Bioinformatics.* 2019.
8. Imakaev M, Fudenberg G, McCord RP, Naumova N, Goloborodko A, Lajoie BR, et al. Iterative correction of Hi-C data reveals hallmarks of chromosome organization. *Nat Methods.* 2012;9(10):999-1003.
9. Schwarzer W, Abdennur N, Goloborodko A, Pekowska A, Fudenberg G, Loe-Mie Y, et al. Two independent modes of chromatin organization revealed by cohesin removal. *Nature.* 2017;551(7678):51-6.
10. Gassler J, Brandao HB, Imakaev M, Flyamer IM, Ladstatter S, Bickmore WA, et al. A mechanism of cohesin-dependent loop extrusion organizes zygotic genome architecture. *EMBO J.* 2017;36(24):3600-18.
11. Crane E, Bian Q, McCord RP, Lajoie BR, Wheeler BS, Ralston EJ, et al. Condensin-driven remodelling of X chromosome topology during dosage compensation. *Nature.* 2015;523(7559):240-4.
12. Jaiswal S, Fontanillas P, Flannick J, Manning A, Grauman PV, Mar BG, et al. Age-related clonal hematopoiesis associated with adverse outcomes. *N Engl J Med.* 2014;371(26):2488-98.
13. Rappsilber J, Mann M, and Ishihama Y. Protocol for micro-purification, enrichment, pre-fractionation and storage of peptides for proteomics using StageTips. *Nature protocols.* 2007;2(8):1896-906.
14. Ritchie ME, Phipson B, Wu D, Hu Y, Law CW, Shi W, et al. limma powers differential expression analyses for RNA-sequencing and microarray studies. *Nucleic Acids Res.* 2015;43(7):e47.

15. Meyers RM, Bryan JG, McFarland JM, Weir BA, Sizemore AE, Xu H, et al. Computational correction of copy number effect improves specificity of CRISPR-Cas9 essentiality screens in cancer cells. *Nature genetics.* 2017;49(12):1779-84.

16. Li W, Xu H, Xiao T, Cong L, Love MI, Zhang F, et al. MAGeCK enables robust identification of essential genes from genome-scale CRISPR/Cas9 knockout screens. *Genome biology.* 2014;15(12):554.

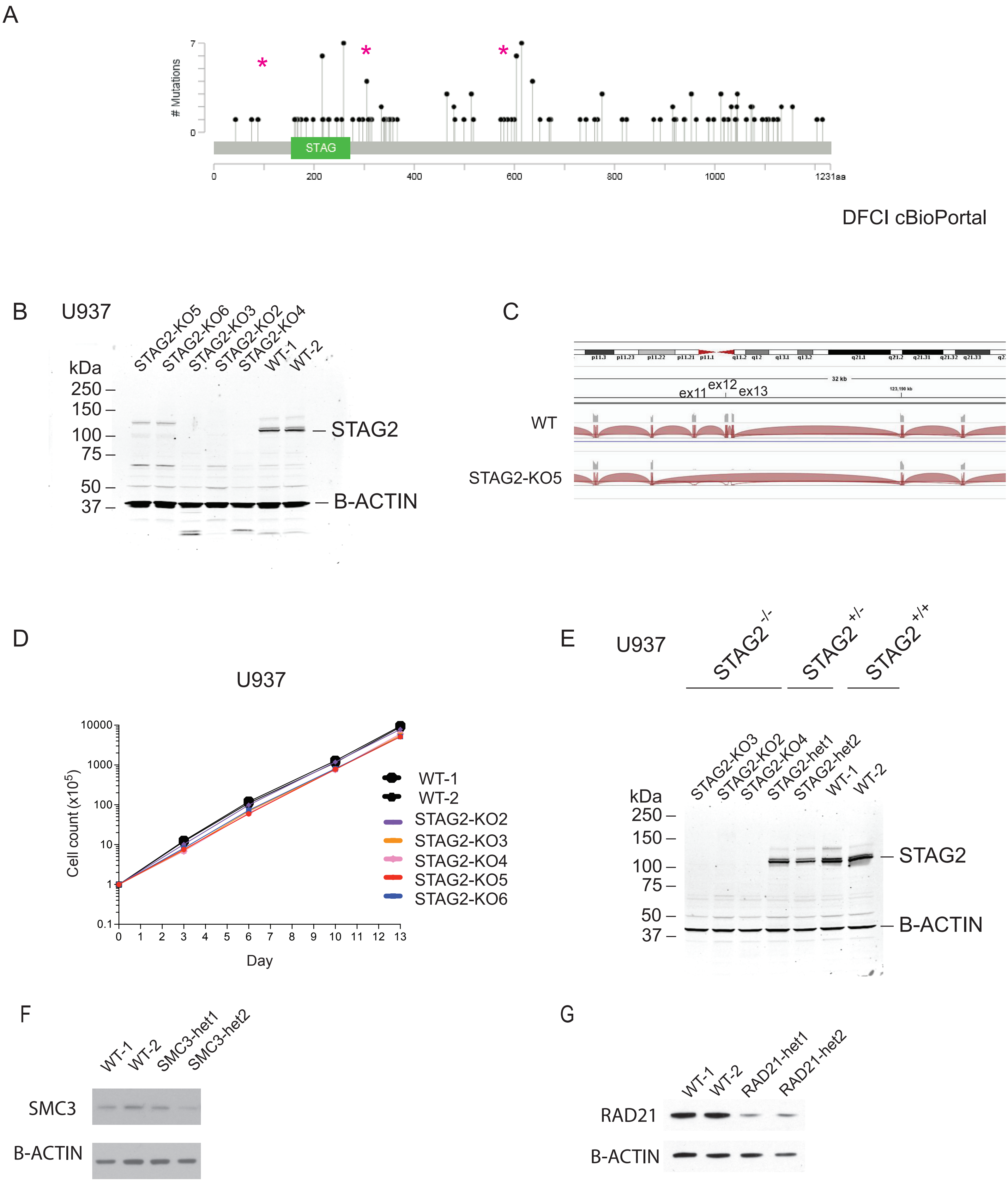
A
Mutations
STAG
DFCI cBioPortal
B
U937
STAG2-KO5
STAG2-KO6
STAG2-KO3
STAG2-KO2
STAG2-KO4
WT-1
WT-2
kDa
STAG2
B-ACTIN
C
ex11
ex12
ex13
WT
STAG2-KO5
D
U937
Cell count (x10^5)
Day
WT-1
WT-2
STAG2-KO2
STAG2-KO3
STAG2-KO4
STAG2-KO5
STAG2-KO6
E
U937
STAG2 -/-
STAG2 +/-
STAG2 +/+
STAG2-KO3
STAG2-KO2
STAG2-KO4
STAG2-het1
STAG2-het2
WT-1
WT-2
kDa
STAG2
B-ACTIN
F
WT-1
WT-2
SMC3-het1
SMC3-het2
SMC3
B-ACTIN
G
WT-1
WT-2
RAD21-het1
RAD21-het2
RAD21
B-ACTIN

Figure S1.

Supplemental Figure S1. Characterization of cohesin-mutant clones used in the study

A, Distribution of presumed pathogenic *STAG2* mutations in patients with hematologic malignancies sequenced at the Dana-Farber Cancer Institute as part of the Rapid Heme Panel (56). Red asterisks mark sites targeted by sgRNAs in this study. Complete list of cell lines generated in this study is listed in the STAR Methods section.

B, Western blotting for STAG2 protein across different U937 cell line clones (expected size 141kDa). β-actin is used as a loading control (expected size 45kDa).

C, IGV track depicting a splicing event affecting exons 11, 12 and 13 of *STAG2* in the U937 STAG2-KO5 clone detected by RNA-sequencing.

D, Growth curve comparing growth characteristics of different U937 *STAG2*-knockout clones.

E, Western blotting for STAG2 protein in U937 cell lines (expected size 141kDa), including heterozygous *STAG2*-mutant cell lines. β-actin is used as a loading control (expected size 45kDa).

F, Western blotting for SMC3 protein in U937 cell lines (expected size 140kDa). β-actin is used as a loading control (expected size 45kDa).

G, Western blotting for RAD21 protein in K562 cell lines (expected size 100kDa). β-actin is used as a loading control (expected size 45kDa).

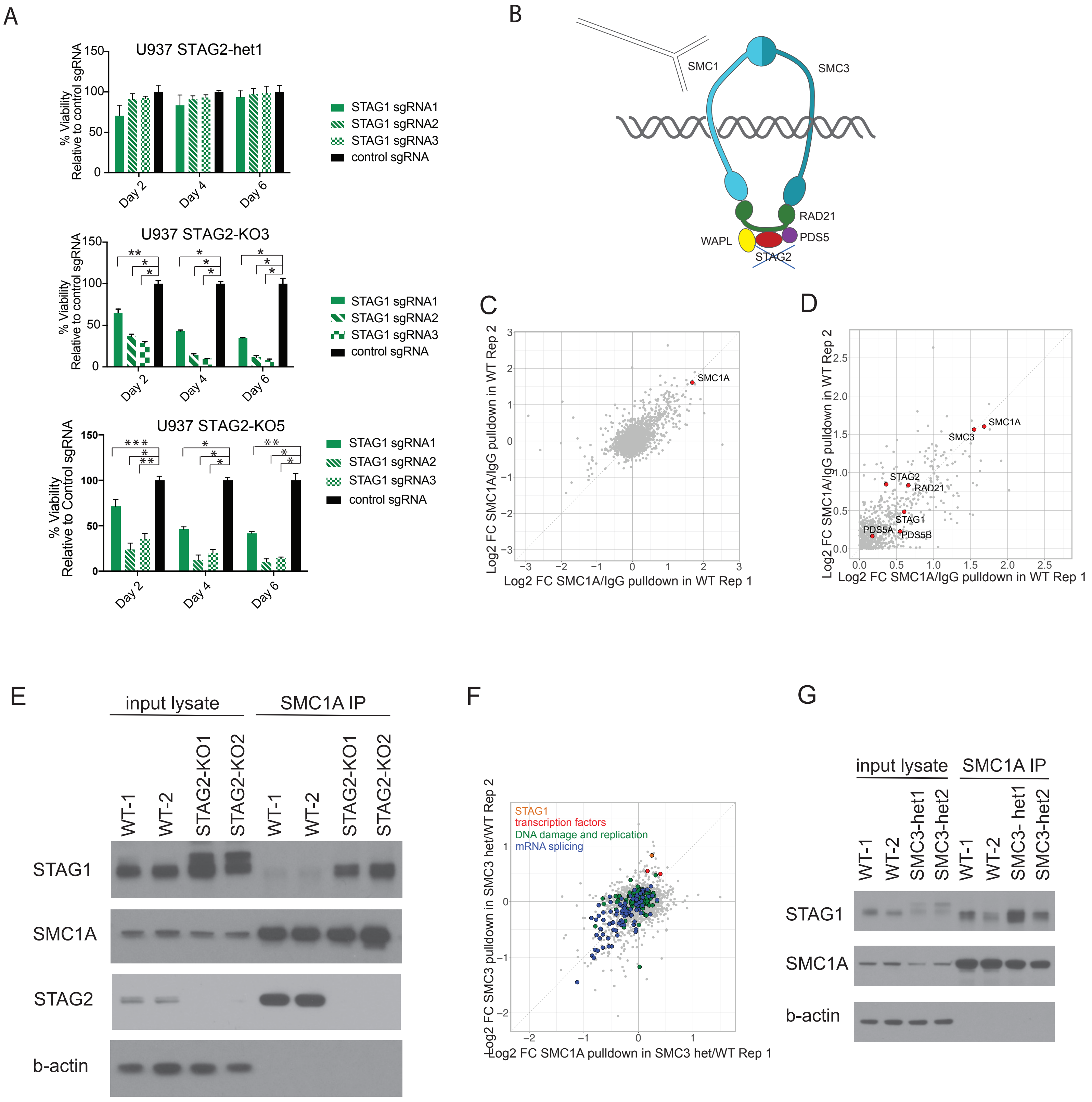
A
U937 STAG2-het1
% Viability Relative to control sgRNA
Day 2
Day 4
Day 6
STAG1 sgRNA1
STAG1 sgRNA2
STAG1 sgRNA3
control sgRNA
U937 STAG2-KO3
U937 STAG2-KO5
% Viability Relative to Control sgRNA
B
SMC1
SMC3
RAD21
PDS5
WAPL
STAG2
C
Log2 FC SMC1A/IgG pulldown in WT Rep 2
Log2 FC SMC1A/IgG pulldown in WT Rep 1
SMC1A
D
SMC3
STAG2
RAD21
STAG1
PDS5A
PDS5B
E
input lysate
SMC1A IP
WT-1
WT-2
STAG2-KO1
STAG2-KO2
STAG1
SMC1A
STAG2
b-actin
F
STAG1
transcription factors
DNA damage and replication
mRNA splicing
Log2 FC SMC3 pulldown in SMC3 het/WT Rep 2
Log2 FC SMC1A pulldown in SMC3 het/WT Rep 1
G
SMC3-het1
SMC3-het2
SMC3- het1

Figure S2.

Supplemental Figure S2. Validation of STAG1 dependency and characterization of the cohesin complex in WT, *STAG2-* and *SMC3*-mutant cells using IP-MS

A, Validation of synthetic lethality between STAG1 and STAG2 loss using CRISPR knockout of *STAG1* in *STAG2+/-* (U937 STAG2-het1) and *STAG2-/-* cells (U937 STAG2-KO3 and KO5). Three independent STAG1 sgRNAs and a non-targeting (NT) sgRNA were used. Mean +/- SD is shown. ** denotes p<0.001, * denotes p<0.0001 (Student t-test)

B, Model depicting members of the cohesin complex and the experimental approach employed for SMC1A immunoprecipitation followed by quantitative mass spectrometry (IP-MS) in wild type (WT) and *STAG2*-knockout AML cells.

C, Scatter plot depicting Log2 fold change (FC) of protein enrichment after SMC1A IP vs IgG IP followed by mass spectrometry in WT U937 cells. Rep 1 and Rep 2 correspond to U937 WT-1 and U937 WT-2 cell lines. Red dot highlights the bait (SMC1A protein).

D, Scatter plot depicting Log2 fold change (FC) of protein enrichment after SMC1A IP vs IgG IP followed by mass spectrometry in WT U937 cells. Rep 1 and Rep 2 correspond to U937 WT-1 and U937 WT-2 cell lines. Red dots highlight different subunits of the cohesin complex.

E, Validation of STAG1 incorporation into the cohesin complex in *STAG2*-knockout cells using immunoprecipitation with SMC1A-specific antibody followed by Western blotting for STAG1, SMC1A, STAG2 and β-actin. U937 WT-1, WT-2, STAG2-KO1 and STAG2-KO2 were used in this experiment. β-actin was used as a loading control.

F, Log2 fold change (FC) of protein enrichment after SMC1A IP-MS in WT and *SMC3*-mutant (57) cells. Rep1 and Rep2 correspond to U937 WT-1 and WT-2, and SMC3-het1 and SMC3-het2 cell lines. Proteins belonging to each gene set are highlighted in color.

G, Validation of STAG1 incorporation into the mutant cohesin complex in *SMC3*-mutant cells using immunoprecipitation with SMC1A-specific antibody followed by Western blotting for STAG1(expected size 144kDa), SMC1A (expected size 143 kDa) and β-actin (expected size 45 kDa). β-actin is used as a loading control.

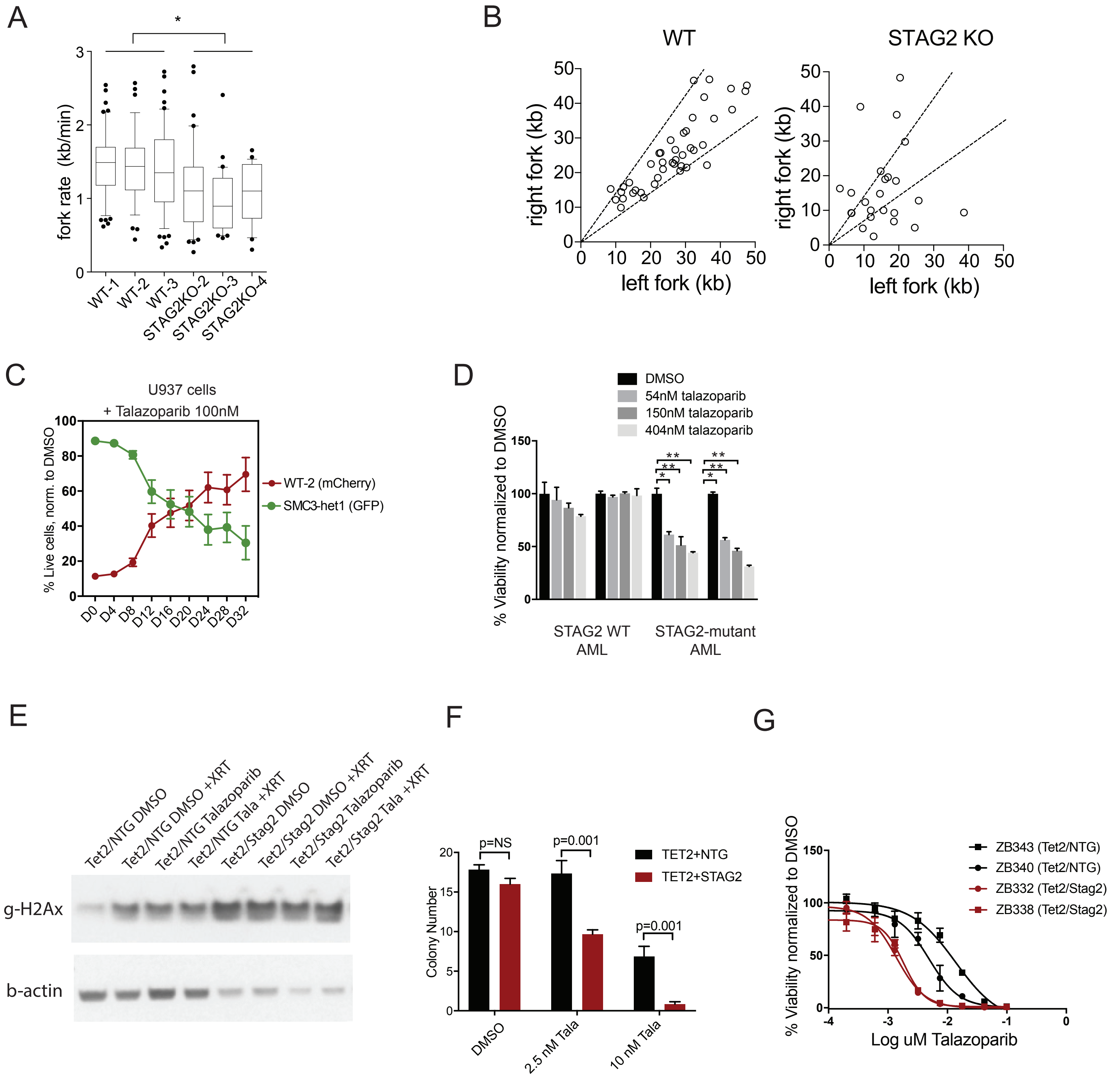
A
*
fork rate (kb/min)
WT-1
WT-2
WT-3
STAG2KO-2
STAG2KO-3
STAG2KO-4
B
WT
STAG2 KO
right fork (kb)
left fork (kb)
C
U937 cells
+ Talazoparib 100nM
% Live cells, norm. to DMSO
WT-2 (mCherry)
SMC3-het1 (GFP)
D0
D4
D8
D12
D16
D20
D24
D28
D32
D
DMSO
54nM talazoparib
150nM talazoparib
404nM talazoparib
% Viability normalized to DMSO
STAG2 WT AML
STAG2-mutant AML
E
Tet2/NTG DMSO
Tet2/NTG DMSO +XRT
Tet2/NTG Talazoparib
Tet2/NTG Tala +XRT
Tet2/Stag2 DMSO
Tet2/Stag2 DMSO +XRT
Tet2/Stag2 Talazoparib
Tet2/Stag2 Tala +XRT
g-H2Ax
b-actin
F
p=NS
p=0.001
p=0.001
TET2+NTG
TET2+STAG2
Colony Number
DMSO
2.5 nM Tala
10 nM Tala
G
ZB343 (Tet2/NTG)
ZB340 (Tet2/NTG)
ZB332 (Tet2/Stag2)
ZB338 (Tet2/Stag2)
% Viability normalized to DMSO
Log uM Talazoparib

Figure S3.

Supplemental Figure S3. DNA replication defects and sensitivity of cohesin-mutant cell lines and primary cells to talazoparib.

A, Replication fork rate in WT and *STAG2*-mutant cells. Box represents first to third quartile, vertical line is the median, and whiskers represent 1.5 X inter-quartile range. $p<0.0001$, unpaired Student t-test

B, Quantification of symmetry of replication fork firing in WT and *STAG2*-mutant cells. Data from 3 WT and 3 *STAG2*-mutant clones were combined. The dashed line represents a ratio of 0.7 between the oppositely moving arms (arbitrarily designated as “left” and “right”) of an origin structure. An asymmetric origin is defined by left:right or right:left ratios of <0.7.

C, Competition assay with WT (U937 WT-2-mCherry) and *SMC3*-heterozygous (U937-SMC3-het1-GFP) clones mixed in 1:10 ratio in the presence of DMSO or talazoparib (100nM). % Live GFP+ or mCherry+ cells were determined using flow cytometry, and % live cells treated with talazoparib and normalized to DMSO control were plotted across different timepoints. Error bars represent SD of measurements of three technical replicates.

D, Primary bone marrow aspirates isolated from patients with *STAG2*- wild type and mutant disease were grown in liquid culture supplemented with cytokines in the presence or absence of specified concentrations of talazoparib or DMSO (n=2/group). Viability was determined on Day 6 using cell-titer glow assay. Sample details are included in the methods section.

E, Western blot analysis of protein lysates isolated from bone marrow cells of *Tet2/*NTG and *Tet2/Stag2* animals treated with DMSO or talazoparib *in vivo*. Half of each sample underwent radiation with 100Rads *ex vivo* and lysates were collected 1 hr later. Immunoblotting for g-H2Ax and actin was done.

F, Bone marrow cells isolated from *Tet2*/NTG and *Tet2/Stag2* mice were grown on methylcellulose supplemented with cytokines in the presence or absence of specified concentrations of talazoparib or DMSO. Number of colonies was counted on Day 14 after plating.

G, Drug dose response curves of Tet2/NTG and *Tet2/Stag2*- mutant bone marrow cells treated with talazoparib. IC_{50} (Tet2/NTG) = 1.4 and 1.8nM, IC_{50} (Tet2/Stag2) = 4.9 and 13.8nM, on Day 8 of treatment. Error bars represent SD of measurements of three technical replicates. Two biological replicates (different mice) were used for the experiment.

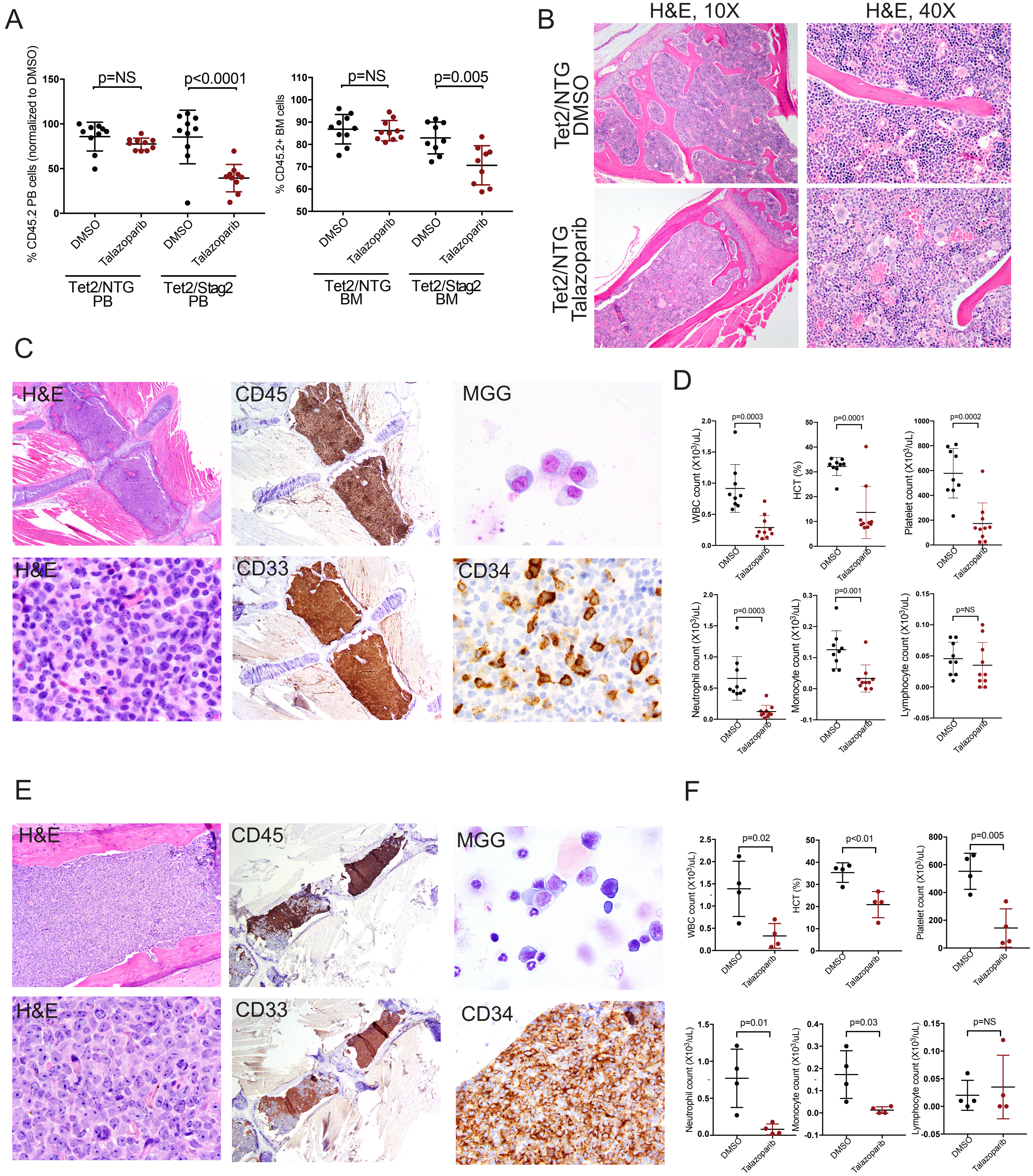

A
p=NS
p<0.0001
% CD45.2 PB cells (normalized to DMSO)
DMSO
Talazoparib
Tet2/NTG PB
Tet2/Stag2 PB
p=NS
p=0.005
% CD45.2+ BM cells
Tet2/NTG BM
Tet2/Stag2 BM
B
H&E, 10X
H&E, 40X
Tet2/NTG DMSO
Tet2/NTG Talazoparib
C
H&E
CD45
MGG
CD33
CD34
D
p=0.0003
p=0.0001
p=0.0002
WBC count (X10³/uL)
HCT (%)
Platelet count (X10³/uL)
p=0.0003
p=0.001
p=NS
Neutrophil count (X10³/uL)
Monocyte count (X10³/uL)
Lymphocyte count (X10³/uL)
E
H&E
CD45
MGG
CD33
CD34
F
p=0.02
p<0.01
p=0.005
p=0.01
p=0.03
p=NS

Figure S4.

Supplemental Figure S4. Development of primary models of cohesin-mutant myeloid disease and *in vivo* testing with talazoparib

A, Peripheral blood flow and bone marrow analysis for presence of donor CD45.2+ cells representing *Tet2*-mutant fraction in *Tet2/*NTG mice and *Tet2/Stag2*-mutant fraction in *Tet2/Stag2* mice at 2 weeks of treatment (peripheral blood) and at 4 weeks of treatment (bone marrow). *Tet2/Stag2* mice show a decrease in mutant cell fraction with talazoparib treatment but there is no effect on *Tet2*-only mutant cells. n=10 per arm. Mean +/-SD shown.

B, Morphologic evaluation of bone marrow of representative *Tet2/NTG*mutant mice treated with talazoparib or DMSO shows no appreciable change in megakaryocyte number and absent erythrophagocytosis. Images were stained using H&E and imaged at 10X (scale bar = 0.5mm) and 40X (scale bar = 0.125mm) magnification.

C, Morphologic evaluation of bone marrow section and bone marrow cell cytospin of secondary transplant recipients of the PDX#1 AML model (*STAG2/RUNX1/ASXL1/NRAS*-mutant disease). Staining with H&E, CD45 and CD33 at 4X magnification (scale bar = 1.25mm), and CD34 and MGG (modified Giemsa May-Grunwald stain) at 100X magnification (scale bar = 0.05mm). Bone marrow is infiltrated with immature CD45+ CD33+ and CD34+ blasts.

D, Complete blood count analysis of PDX#1 AML model treated with talazoparib versus vehicle after 4 weeks of treatment. Talazoparib treated animals show reduction in white blood cell (WBC) count, neutrophil count, hematocrit (HCT) and platelet count. n=9-10 per arm. Mean +/-SD shown.

E, Morphologic evaluation of bone marrow section and bone marrow cell cytospin of secondary transplant recipients of the PDX#2 AML model (*RAD21/RUNX1/WT1/FLT3*-mutant disease). Staining with H&E, CD45 and CD33 at 4X magnification (scale bar = 1.25mm), and CD34 and MGG (modified Giemsa May-Grunwald stain) at 100X magnification (scale bar = 0.05mm). Bone marrow is infiltrated with immature CD45+ CD33+ and CD34+ blasts.

F, Complete blood count analysis of PDX#1 AML model treated with talazoparib versus vehicle after 2 weeks of treatment. Talazoparib treated animals show reduction in white blood cell (WBC) count, neutrophil count, hematocrit (HCT) and platelet count. n=4 per arm. Mean +/-SD shown.

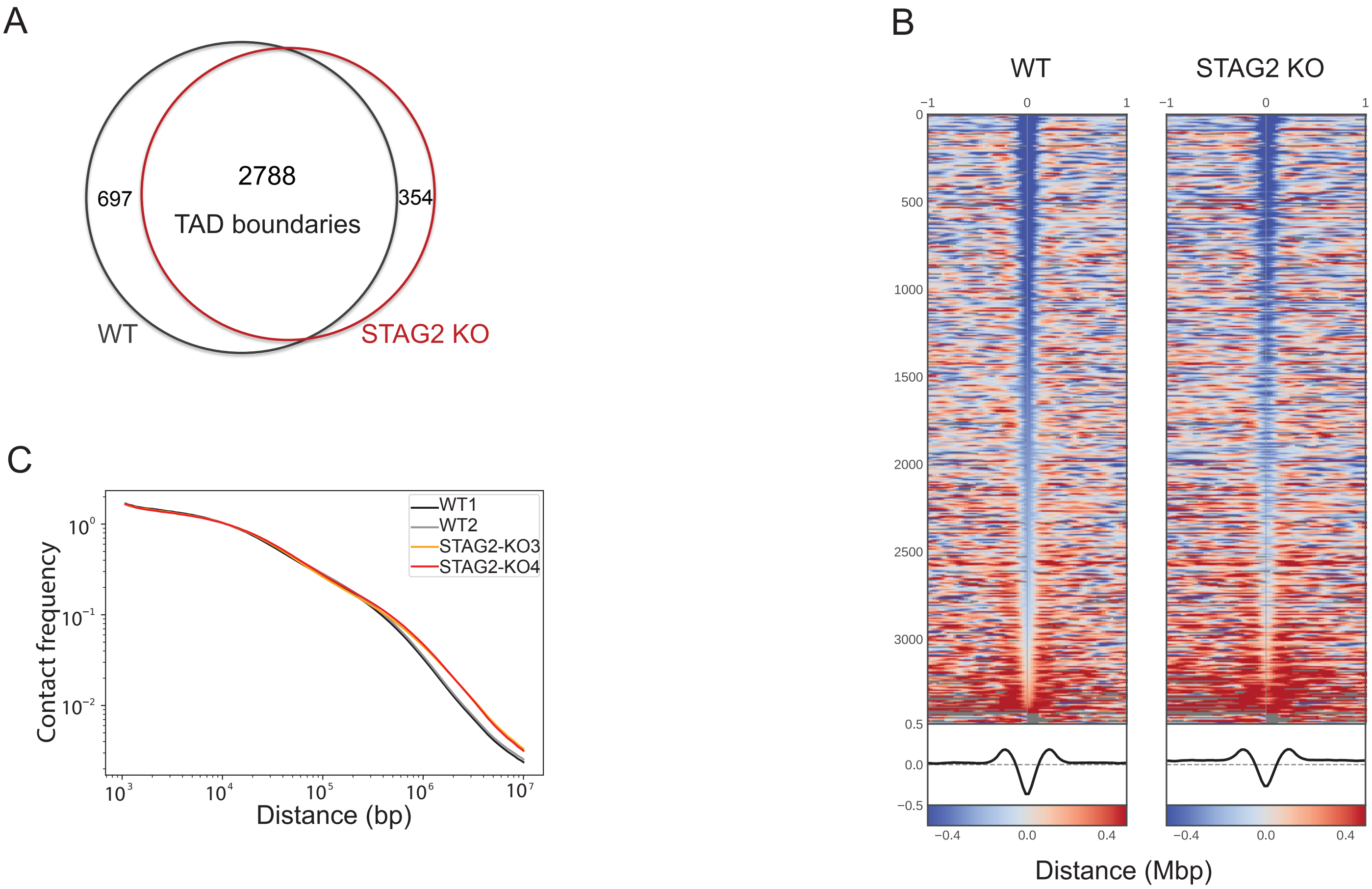
A
2788
TAD boundaries
697
354
WT
STAG2 KO
B
WT
STAG2 KO
−1
0
1
500
1000
1500
2000
2500
3000
0.5
0.0
−0.5
−0.4
0.0
0.4
Distance (Mbp)
C
WT1
WT2
STAG2-KO3
STAG2-KO4
Contact frequency
10^0
10^{-1}
10^{-2}
10^3
10^4
10^5
10^6
10^7
Distance (bp)

Figure S5.

Supplemental Figure S5. STAG2 loss alters chromatin organization and leads to longer loop extrusion

A, Venn diagram depicting overlap of conserved TAD boundaries between WT and STAG2 KO cells. Common TAD boundaries with an insulation strength >0.3 were determined for the two WT (3485) and STAG2 KO (3142) biological replicates. 2788 TAD boundaries are shared by WT and STAG2 KO cells.

B, Stacked heatmaps of insulation scores at WT boundaries. There is a global loss of insulation in STAG2 KO cells.

C, Scaling plots representing the genome wide contact frequency as a function of genomic separation for all intra-chromosomal interactions in WT (U937 GC1-A1, U937 GC3-E1) and STAG2 KO (U937 STAG2-KO3, U937-STAG2-KO4) cells. The scaling plots were normalized to unity at separation = 100 kb.

SUPPLEMENTAI TABLE LEGENDS

Supplemental Table S1. Differential dependency analysis of genome-wide CRISPR screens in *STAG2*-mutant and wild type U937 cells.

Significance of differential gene dependencies between *STAG2*-mutant and *STAG2*-wild type U937 cell lines were assessed using the MLE method from MAGeCK version 0.5.6.

Supplemental Table S2. Proteomics analysis of *STAG2*- and *SMC3*-mutant U937 cell lines.

a, SMC1A vs IgG IP-MS in *STAG2* WT U937 cells
b, SMC1A IP-MS in *STAG2* KO vs WT U937 cells
c, SMC1A IP-MS in *SMC3*-mutant vs WT U937 cells

Supplemental Table S3. Single cell clones used in the study.

SUPPLEMENTAL DATA SETS

Genome-wide CRISPR screen and analysis: Raw and processed data have been deposited at figshare
DOI [10.6084/m9.figshare.7119749; reviewer access at https://figshare.com/s/893848c38e01a5deeec3] and
DOI [10.6084/m9.figshare.7120796; reviewer access at https://figshare.com/s/47ea327476fc114f853f]

Proteomics: The original mass spectra have been deposited in the public proteomics repository MassIVE and are accessible to reviewers at ftp://MSV000082970@massive.ucsd.edu when providing the dataset password: leukemia. If requested, also provide the username: MSV000082970. This data will be made public upon acceptance of the manuscript.

Supplemental Data Table 3: Single cell clones used in the study

Clone ID	Full clone name	Genotype	Experiments used
**U937 cells**			
WT-1	U937-GC1-A1	wild type	IP-MS, CRISPR screen, Hi-C, chromosome spreads, replication fork stability, DNA damage analysis, STAG1 synthetic lethality, drug testing
WT-2	U937-GC3-E1	wild type	IP-MS, CRISPR screen, Hi-C, chromosome spreads, replication fork stability, DNA damage analysis, STAG1 synthetic lethality, drug testing
WT-3	U937-GC3-E3	wild type	replication fork stability, drug testing
WT-4	U937-GC3-E1	wild type	drug testing
STAG2-KO1	U937-STAG2-A2-F8	STAG2 exon 17, 68bp deletion/54 bp deletion	IP-MS, DNA damage analysis, drug testing
STAG2-KO2	U937-STAG2-A2-G4	STAG2 exon 17, 1bp insertion, homozygous	IP-MS, replication fork stability, DNA damage analysis, drug testing
STAG2-KO3	U937-STAG2-A2-F11	STAG2 exon 17, 1bp deletion, homozygous	IP-MS, CRISPR screen, Hi-C, chromosome spreads, replication fork stability, DNA damage analysis, drug testing
STAG2-KO4	U937-STAG2-A2-H4	STAG2 exon 17, 19bp deletion, homozygous	IP-MS, CRISPR screen, Hi-C, chromosome spreads, replication fork stability, DNA damage analysis, drug testing
STAG2-KO5	U937-STAG2-A1-C4	STAG2 exon 12, 1bp insertion, homozygous	IP-MS, DNA damage analysis, drug testing
STAG2-KO6	U937-STAG2-A1-D10	STAG2 exon 12, 1bp insertion, homozygous	IP-MS, DNA damage analysis, drug testing
STAG2-het1	U937-STAG2-A1-A3	STAG2 exon 12, 1 bp deletion, 1 WT	STAG1 synthetic lethality
STAG2-het2	U937-STAG2-A2-H3	STAG2 exon 17, 1 bp deletion, 1 WT	STAG1 synthetic lethality
NCB1	U937-Cas9 GC1-B1	wild type	CRISPR screen
NCB12	U937-Cas9 GC1-B12	wild type	CRISPR screen
NCB2A	U937-Cas9 GC1-B2(A)	wild type	CRISPR screen
NCC4	U937-Cas9 GC1-C4	wild type	CRISPR screen
KOC5	U937-Cas9 STAG2-C5(C)	STAG2 exon 4, 1bp insertion, homozygous	CRISPR screen
KOD5C	U937-Cas9 STAG2-D5(C)	STAG2 exon 4, 1bp insertion, homozygous	CRISPR screen
KOG8B	U937-Cas9 STAG2-G8(B)	STAG2 exon 4, 68bp deletion, homozygous	CRISPR screen
SMC3 het 1	U937-Cas9-SMC3-H8	SMC3 exon 9, 27 bp deletion, WT	IP-MS, drug testing
SMC3 het 2	U937-Cas9-SMC3-H10	SMC3 exon 9, 17 bp del, 15 bp del, 1bp substitution	IP-MS, drug testing
**K562 cells**			
WT-1	K562-GC1-a7	wild type	drug testing
WT-2	K562-GC1-a1	wild type	drug testing
STAG2	K562-STAG2-A1-a3	STAG2 exon 12, 1bp deletion, 9bp deletion	drug testing
SMC3 het	K562-SMC3-G5-e4	SMC3 exon 9, 1bp insertion, WT	drug testing
RAD21	K562-RAD21-G2-b8	RAD21 exon 2, 2bp deletion, WT	drug testing

Figure 1E - gH2Ax (Lanes 1-8)

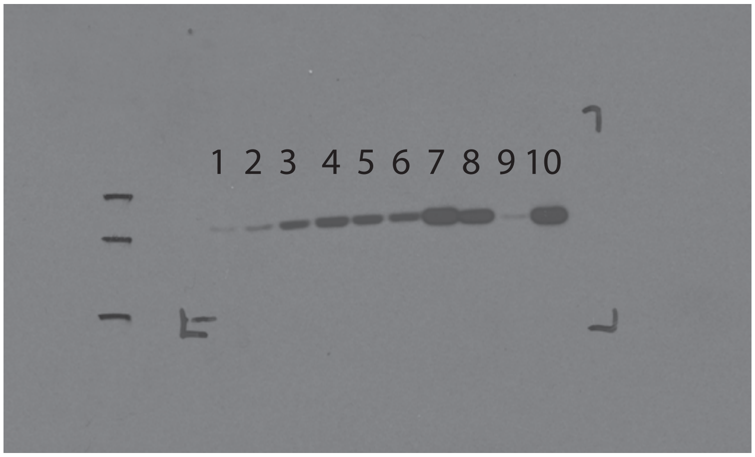

Figure 1E - b-actin (Lanes 1-8)

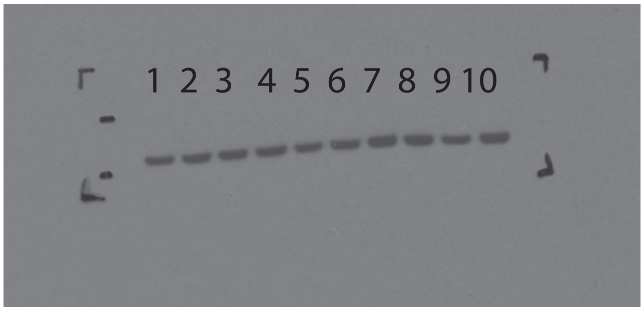

Figure 1F - vinculin (Lanes 1-4, 9-12)

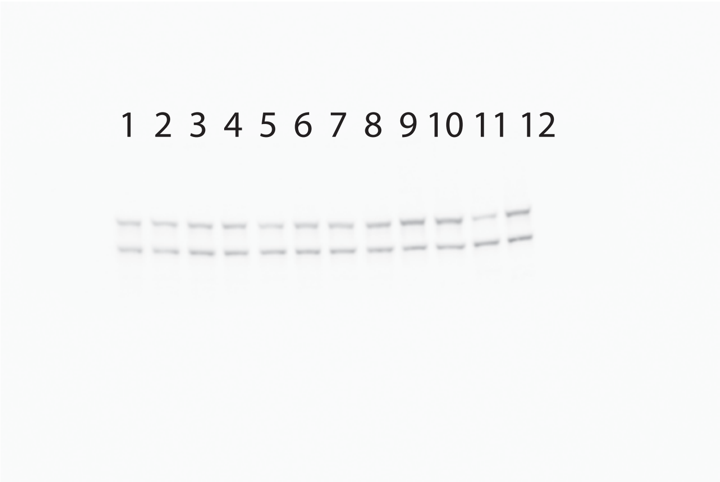

Figure 1F - pATM, pATR (Lanes 1-4, 9-12)

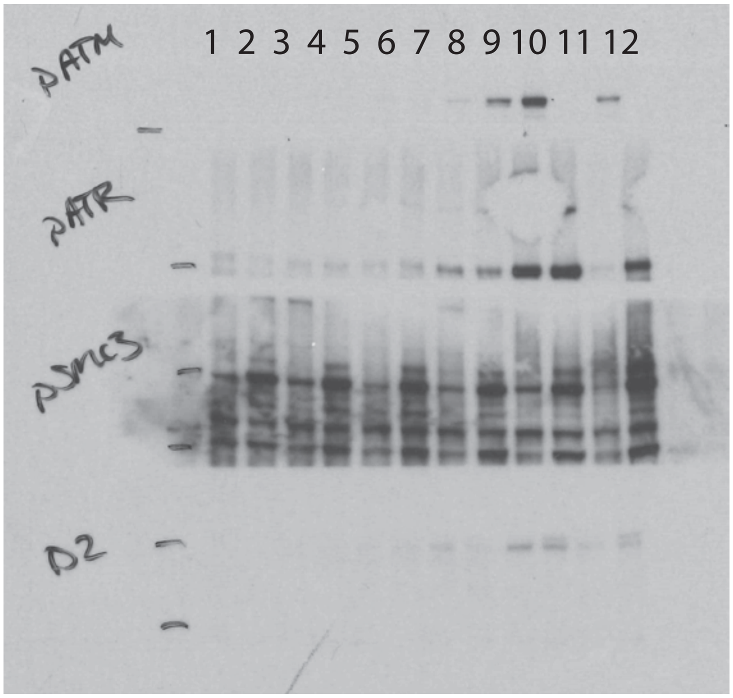

Figure 1F - ATM (Lanes 1-4, 9-12)

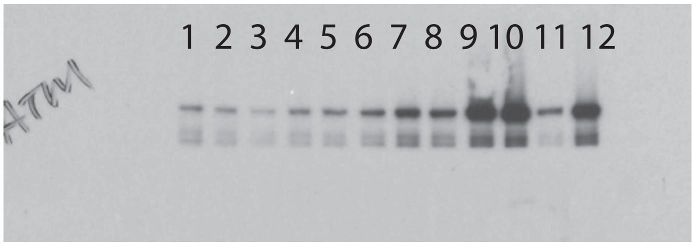

Figure 1F - ATR (Lanes 1-4, 9-12)

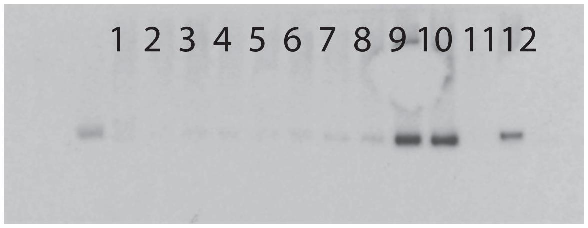

Figure 1F - pCHK1 (Lanes 1-4, 9-12)

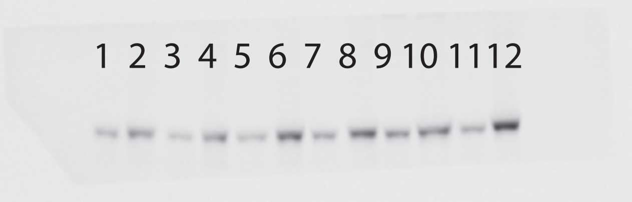

Figure 1F - CHK1 (Lanes 1-4, 9-12)

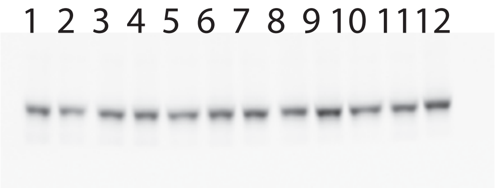

Figure 1F - pCHK2 (Lanes 1-4, 9-12)

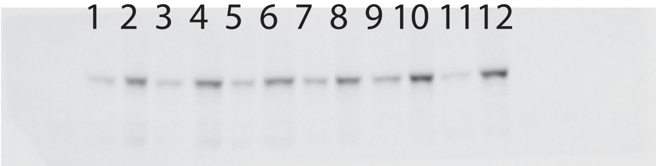

Figure 1F - CHK2 (Lanes 1-4, 9-12)

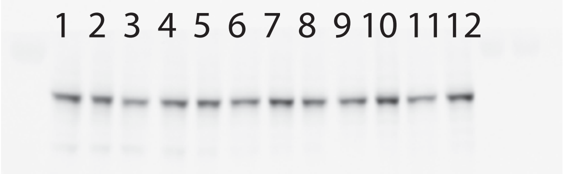

Figure S1B - STAG2 and b-actin (Lycor)
(Lanes 1-7)

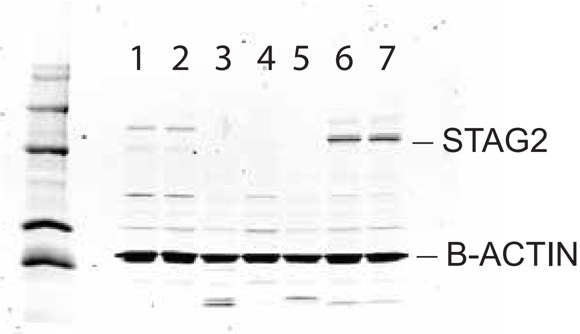

Figure S1E - STAG2 and b-actin (Lycor)
(Lanes 1-7)

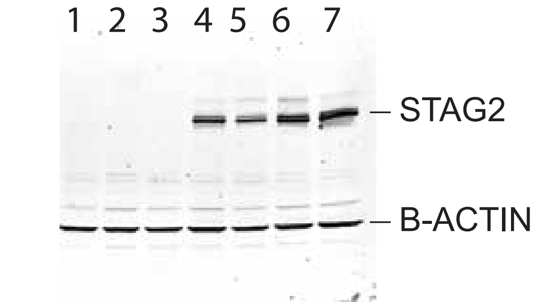

Figure S1F - SMC3 (Lanes 1-4)

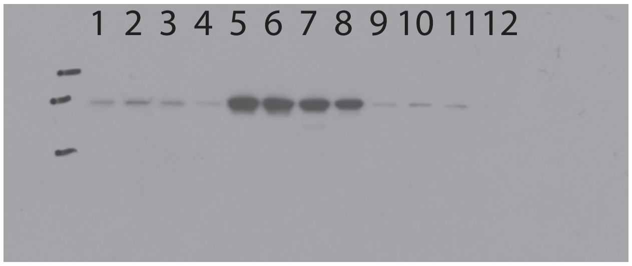

Figure S1F - b-actin (Lanes 1-4)

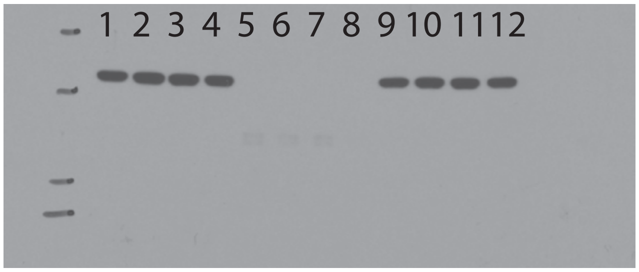

Figure S1G - RAD21 (Lanes 1-4)

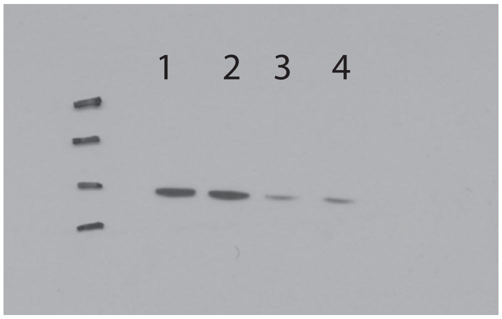

Figure S1G - b-actin (Lanes 1-4)

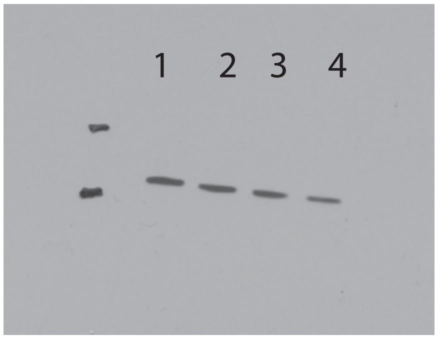

Figure S2E - STAG1 (Lanes 1-8)

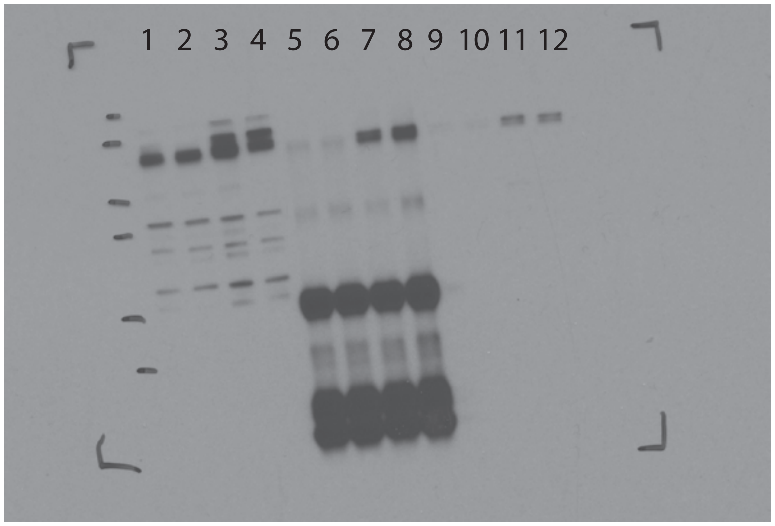

Figure S2E - SMC1A (Lanes 1-8)

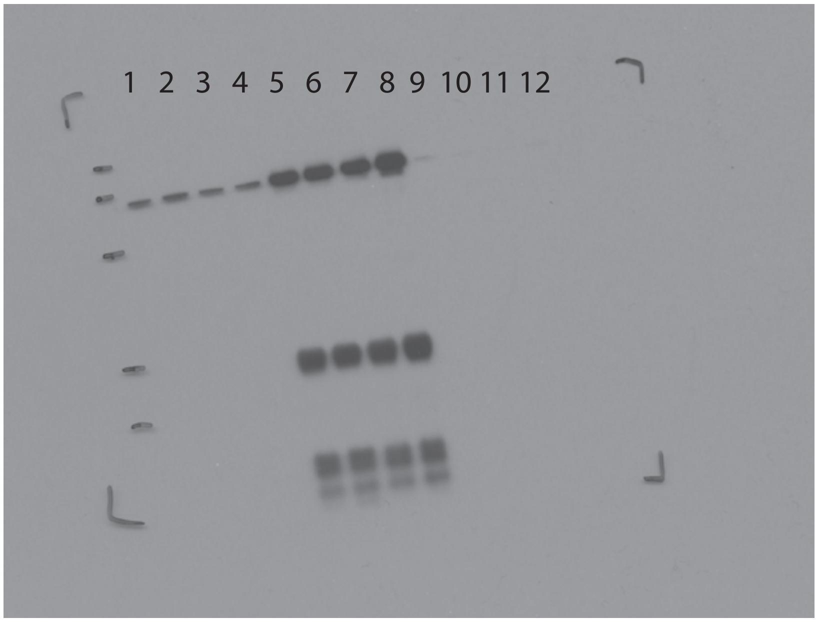

Figure S2E - STAG2 (Lanes 1-8)

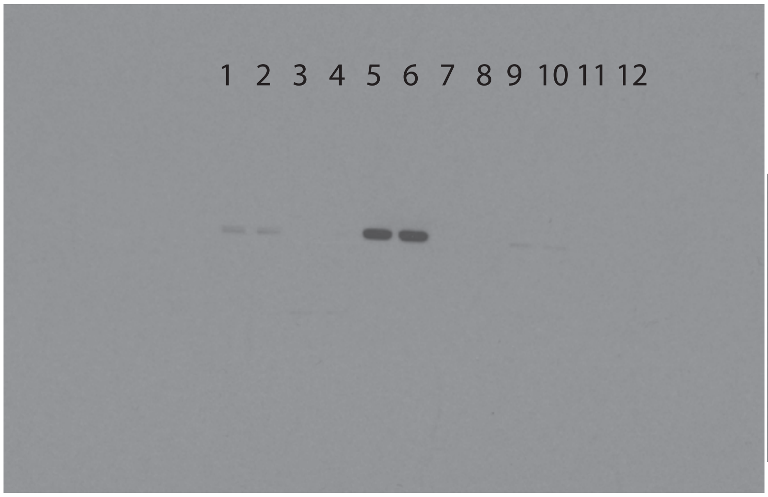

Figure S2E - b-actin (Lanes 1-8)

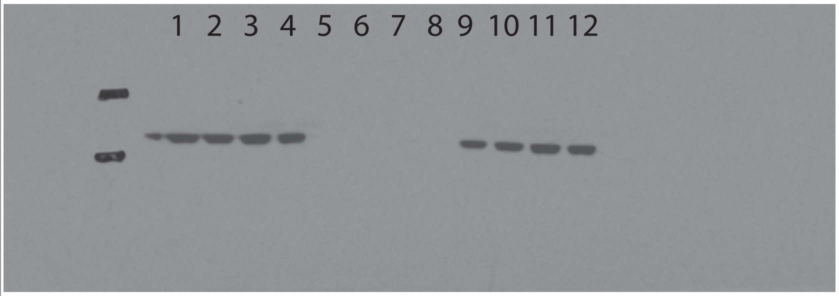

Figure S2G - STAG1 (Lanes 1-8)

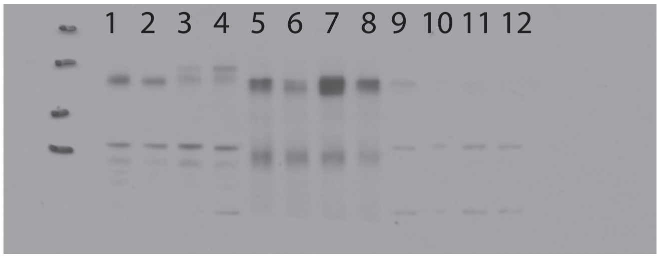

Figure S2G - SMC1A (Lanes 1-8)

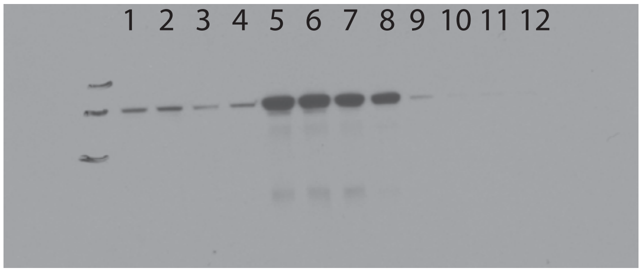

Figure S2G - b-actin (Lanes 1-8)

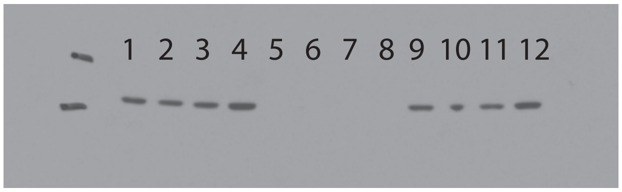

Figure S3E - gH2Ax, b-actin (Lanes 1-8)

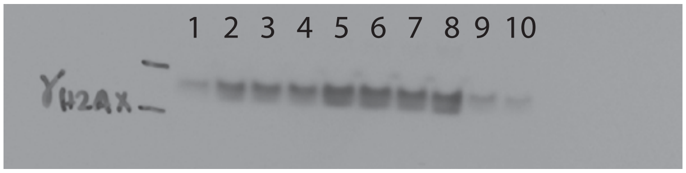

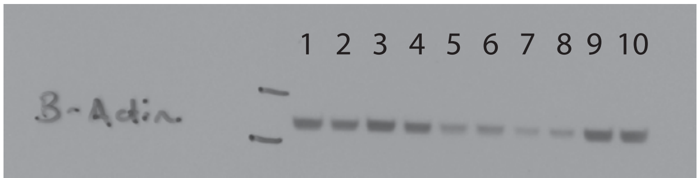

Supplement: Supplemental data [file jciinsight-6-142149-s252.pdf]
